# Time series analysis of malaria in pregnancy, using wavelet and SARIMAX models

**DOI:** 10.1371/journal.pone.0328888

**Published:** 2025-08-06

**Authors:** Dolapo Oluwaseun Oniyelu, Olaiya Folorunsho, Lawrence Adewole, Emmanuel Afolabi Bakare, Chukwu Okoronkwo, Nelson Eze, with ICAMMDA Team

**Affiliations:** 1 Department of Computer Science, Federal University Oye-Ekiti, Ekiti State, Nigeria; 2 International Centre for Applied Mathematical Modelling and Data Analytics (ICAMMDA), Federal University Oye-Ekiti, Ekiti State, Nigeria; 3 Unit for Data Science and Computing, North West University, Potchcefstroom, South Africa; 4 Department of Mathematics, Federal University Oye-Ekiti, Ekiti State, Nigeria; 5 National Malaria Elimination Programme (NMEP), Abuja, FCT Nigeria, Nigeria; University of Uyo, NIGERIA

## Abstract

Malaria in pregnancy (MIP) remains a global health challenge, affecting approximately 40% of pregnant women. Despite malaria control efforts by the Nigerian Government and its partners, regional disparities in health outcomes and malaria incidence trends among pregnant women remain under-studied. This study objectives were to assess MIP variability compared to general malaria cases, and forecast short-term MIP incidence over two years. This was achieved by analyzing malaria in pregnancy (MIP) variability across Nigeria from January 2015 to January 2025, using wavelet coherence, patterns of transmission cycles and selecting best modelling approach by comparing ARIMA and SARIMAX models to assess temporal trends before the forecast of short-term MIP incidence. Findings showed significant regional variability, with Cross River peaking in 2017 and 2019, while Enugu recorded its lowest trough in 2017. Malaria peaks in southern states remained lower than troughs in northern regions. Strong cross-correlations between MIP and general malaria transmission cycles were observed in Kebbi, Niger, Yobe, and Ondo, indicating persistent trends, while South-South and South-East exhibited weaker correlations, likely due to intervention fluctuations. SARIMAX models captured MIP trends more effectively, except Kebbi, where ARIMA fit better, and Niger, where SARIMAX exaggerated forecasts due to sensitivity to exogenous variables. Thus, SARIMAX was adopted for Cross River, Enugu, Ondo, and Yobe; while ARIMA was used for Kebbi and Niger States. It was discovered that Cross River and Enugu exhibited intervention-driven malaria fluctuations, Ondo, Niger, and Yobe displayed unstable or cyclical trends, reinforcing the importance of climate-sensitive forecasting models and seasonal interventions for improving malaria prediction accuracy. South-South and South-East need improved healthcare access, North-Central and North-West require seasonality forecasting, while North-East demands urgent control measures. Targeted malaria interventions are crucial to support achievement of the Nigeria’s National Malaria Elimination Programme (NMEP) goals.

## Introduction

Malaria is an endemic disease with high prevalence in Sub-Saharan Africa, where Nigeria carries the highest burden [1,2]. It is a parasitic disease that is transmitted by infected female Anopheles mosquitoes during their blood meal. This is common in endemic regions due to the favourable climatic conditions with the prevalence of *Plasmodium falciparium* being the major vector specie particularly in the Nigeria [3]. According to [4], there is about 40% possibility of exposure to malaria in pregnancy (MIP) in the world. World Health Organization (WHO), recommended that interventions including: intermittent preventive treatment with sulfadoxine-pyrimethamine (IPTp-SP) and long-lasting insecticidal treated net (LLIN) be used in the control of MIP [5,6]. In Nigeria, the last decade of malaria control has witnessed a huge increase in effort by the Government and its partners towards the up-scaling of key interventions such as mass campaigns for replacement of insecticide-treated nets (ITNs), intermittent preventive treatment of malaria during pregnancy (IPTp) and malaria case management [7]. Malaria is a major public health challenge, with Nigeria accounting for nearly 27% of the global malaria burden [8,9], which is a significant component of the global malaria burden. Pregnant women still remain one of the most vulnerable populations, despite substantial efforts to reduce the prevalence of malaria [7] among this population. The variability in the incidence of malaria differs based on varying impact of malaria and the peculiarity of different geographic locations, making the elimination of malaria specific for different regions of the country [10]. Nigeria has different climate and topography with uplands (600 to 1,300 meters in the North Central Zone), lowlands (less than 20 meters in the coastal areas) and highlands in the eastern parts [11].

The Nigerian Government commenced the deployment of LLIN since 2009 [12] and IPTp in 2005 [13]. According to the 2021 Nigeria Malaria Indicator Survey (MIS), it was reported that regarding the use of ITNs by pregnant women, 50% of pregnant women aged 15–49 were reported to have slept under an ITN the night before the survey [7]. On the other hand, 31% of women aged 15–49 with a live birth in the 2 years preceding the survey reported taking three or more doses of sulfadoxine-pyrimethamine (SP) during their last pregnancy [7]. It was observed by [14] that more than 120 million pregnancies from 2010 to 2020 in malaria-endemic parts of the world, recorded an annual infant mortality rate ranging between 75,000 and 200,000. Pregnant women who took SP twice or three times recorded lower incidence of malaria in their fetus than those who took it only once [15,16]. MIS reported that the usage of IPTp-SP among the vulnerable pregnant women population improved from 34% to 50% in 2021 [7]. Although several study on some States of the country have been carried out on efforts to reduce malaria, particularly during pregnancy in Nigeria [17–21], little is known about the role that regional variations played on health outcomes of MIP incidence and the temporal pattern over the years in the varying regions. MIP is strongly associated with infant low birth weight due to prematurity, intrauterine growth restriction (IUGR), maternal anemia and elevated neonatal mortality [22,23].

These factors increase the risk of neonatal morbidity and mortality, highlighting the urgent need for monitoring and evaluation of the progress made on prevention strategies. While malaria control efforts by the Nigerian Government and international health partners have led to improvements, regional disparities in MIP incidence and health outcomes remain insufficiently embarked. Despite the fact that many have studied on general malaria prevalence [24], there exist limited research which have explicitly looked at the regional and temporal patterns in MIP throughout Nigeria. Rather than examining how MIP incidence varies, studies on the efficacy of IPTp coverage and vector control initiatives are not streamlined to MIP transmission [25,26]. This gap presents challenges in designing region-specific interventions to mitigate MIP cases in high-risk zones, which helps targeted interventions and continued evaluation of the effect of malaria on this vulnerable group. There is therefore a need to carry out a pre-analysis of the trends and understand the variability in the patterns of MIP incidence within the 6 regions of Nigeria. The geo-political regions are grouped as: South-Southern, South-Eastern, South-Western, North-Western, North-Central and North-Eastern Nigeria. In this study, each region is represented by a State from the region having one of the highest burden of malaria in that region based on MIS 2021 report. This study seeks to analyze the variability of MIP incidence across these Nigerian regions using time series methods, comparing its trends with general malaria incidence. Forecasting of future incidence of MIP using wavelet coherence to denoise data and selection of best modelling approach by comparing ARIMA and SARIMAX models is also carried out. By translating coherence cycles into real-world malaria transmission periodicity, insight into seasonal malaria trends and role of exogenous factors such as pandemic disruption or intervention is analysed. Understanding short-term fluctuations and seasonality will help refine policy approaches for targeted malaria control programs, ensuring region-specific strategies that align with Nigeria’s National Malaria Elimination Programme (NMEP) goals.

Time series are made up of records or data points collected over a certain time range [27], for which analyses of datasets with a timestamp index can be carried out to obtain the pattern or trend inherent in the data [28]. Analyzed time series data can be used to forecast or predict other data points which could be of great importance in making early decisions for improved health outcomes [29]. Univariate or multivariate time series analysis can be approached using the time domain or the frequency domain [30–32]. Artificial Intelligence (AI) techniques are needed in the public health sector [33] and there is the growing acceptance of Machine Learning (ML) use, in identifying life-threatening diseases early for the improvement of patient survival rates [34–39]. This is demonstrated in the study of [40], which presented artificial intelligence (AI)-inspired techniques to record real-time forecasted COVID-19 and forecast the incidence of various provinces Various literature used diverse approaches including ML and simple statistics-based time series as forecast or predictive models [34,41–44], however, these algorithms are seen as not easily interpretable models and medical practitioners are skeptical about the forecast or predicted outcomes of such models [45]. There is therefore the need for interpretable models or use of eXplainable Artificial Intelligence (XAI) modelling approach. XAI are efforts developed to enable the understanding of the inner AI processes followed to arrive at a models’ outcome [46]. To achieve this, interpretable models such as ARIMA [47], SARIMAX [48] and wavelet models [49] which are time series analysis tools was used in the study to carryout pre-analysis of the trends of MIP. This is also used to gain understanding of the variability inherent in the patterns of various regions in Nigeria.

Time series analysis in the time domain can be achieved using either the auto correlation analysis on univariate data (such as Autoregressive Integrated Moving Average: ARIMA model) or cross correlation analysis on multivariate datasets [50–52]. ARIMA models are based on a combination of past trends (autoregressive portion), past forecast errors (moving average component), and past differencing (integrated part) in the various regions [53]. This facilitates comprehension of the model’s operation and prediction-making process. Time series analysis in the frequency domain can be achieved either by using the spectrum or wavelet transform [54,55]. Wavelet analysis can be considered an interpretable model, providing a clear interpretation in both spatial and frequency domains, making them suitable for many applications [56]. They can be used to analyze time series data to implement denoising of the data and viewing of inherent characteristics of the data in the frequency domain [57,58]. Data denoising is important due to the presence of varying random noise and seasonal changes that could be found in the time series [59].

In [60] work, an hybrid methodology was applied using discrete wavelet decomposition to the death due to COVID ’19 dataset by spliting the input data into component series and then using ARIMA to make predictions. The prediction error of their result was compared to that obtained from an ARIMA model which showed the performance of prediction from hybrid wavelet-ARIMA model to give more accurate result. The limitation of the work however lies in the use of Daubechies wavelet to carry-out the analysis, whose strength is limited to noise reduction and data compression. The work of [61] studied time series forecasting techniques using two hybridization techniques that integrated wavelet ARIMA and GARCH, with discrete Fourier transform for the ARIMA pre-processing. This enhanced the ARIMA forecast by utilizing each model’s capacity to identify both linear and non-linear patterns seen in a time series. They pointed out the need to further rate the effectiveness of this method in other fields. The work of [62], separated feature sets with and without Discrete Wavelet Transform (DWT) to compare deep learning models intended to predict the daily number of COVID-19 incidence and deaths for 183 countries. The results from the homogeneous architecture comprising multiple LSTM (Long-Short Term Memory) layers, and the hybrid architecture merging multiple CNN (Convolutional Neural Network) layers and multiple LSTM layers showed a statistically significant difference between the models’ performances both for the prediction of deaths and confirmed incidence (with p-value <0.001). The CNN+LSTM model performed similarly when wavelet coefficients were included as extra features (DWT+CNN+LSTM), indicating the promise of wavelets as an optimization tool. To forecast short-term trends in confirmed COVID-19 cases across several locations, including the US, Asia, Europe, Africa, and others, [63] used the ARIMA and ARIMAX models. The study also looked at the connection between vaccination rates and the number of new cases, as well as the effects of socioeconomic variables like GDP per capita and healthcare resources on COVID-19 incidence rates in various nations. They however noted the need for further research on the importance of region-specific strategies in understanding the variability in outcomes across different regions. According to [64], the yearly and monthly vaccination patterns of regular childhood immunization programs was assessed while taking the COVID’19 pandemic disruption into account. ARIMA model was used for predictive modelling to show how vaccination rates varied by area and how seasonal variations were seen in monthly vaccination rates, with Bacillus Calmette-Guérin (BCG) vaccine having the most consistent pattern. They were able to get insight on the dynamics of childhood vaccination, although due to lack of historical data (ranged only between: 2016–2018), their observation of long-term patterns was also limited.

To further validate the use of hybrid wavelet-ARIMA model, pre-analysis of data that combines the strength of trend filtering and cross-correlation on time series data while also performing denoising using the morlet wavelet transform algorithm is important. This was carried out in this study on historical data of a long-term observations on MIP cases from January 2015–January 2025), to get better understanding on the nature of the trend and varying patterns of MIP in the various regions of Nigeria. Morlet wavelet whose strength lies in the decomposition of time series to the time-frequency domain was thus proposed to get better insights from MIP time series. An hybrid wavelet model was applied with ARIMA/SARIMAX model to analyse the data and improve forecast results and performance accuracy. This study addressed questions centered on understanding i) what the long term trend in MIP has been from January 2015 till January 2025, in each region of Nigeria, ii) what the variability of MIP incidence over time had been compared to the general malaria incidence (MC) in each region, and iii) how short-term trends of MIP incidence of months to come can best be forecasted. The aim of the study was to carryout a time series pre-analysis of MIP incidence in Nigeria (Jan 2015–Jan 2025) in comparison with the general MC, and provide a 2 year forecast of possible future trends, using an hybrid of wavelet and ARIMA/SARIMAX models. This was carried out within six highest burdened States with each serving as representatives of the six (6) geo-political regions of Nigeria namely: South-Southern (Cross Rivers State), South-Eastern (Enugu State), South-West (Ondo State), North-Western (Kebbi State), North-Central (Niger State), and North Eastern (Yobe States) regions. The specific objectives were to: i) carryout pre-analysis and visualization of MIP and MC long-term trend, ii) analyze the variability of MIP incidence over time compared to general MC incidence, and; iii) forecast a 2 year short term trends of possible future trends of MIP incidence. This would help in gaining valuable insights into the underlying transmission pattern of malaria within the pregnant women population. Generating these forecast for years to come would help support the understanding of the nature of malaria trend in the country in the past and inform planning, preparedness and decision making of the public health sector of Nigeria, in the enrollment of control in reducing the further spread of malaria endemicity in Nigeria.

Wavelet techniques have been effectively used in many fields and building on the strength of both wavelet and ARIMA as an hybrid model [44,61], this study pre-analyses MIP incidence data of Nigeria regions, represented by six states. The wavelet-ARIMA hybrid combines the strengths of both approaches by using wavelet transformation to enhance noise reduction and improve prediction accuracy. Wavelets are used as optimization tool to achieve enhanced preprocessing of the time series dataset and improve the understanding of the seasonal variability present in the historical data of MC and MIP counts. In achieving the ascertainment of the effectiveness of malaria control efforts in Nigeria, especially in pregnancy, and understanding of the temporal pattern over time in various regions of the country; the ARIMA and Wavelet hybrid modelling approach was adopted. This will help the pre-analysis of MIP monthly incidence time series data, using a 10 years historical data in gaining insight to long-term patterns in six selected hotspot states, representative of the six geo-political zones in Nigeria. The achievement of great accuracy in the monitoring of the progression of malaria would enable strategic planning towards to ultimately enhance monitoring and surveillance strategies of the public healthcare systems in attaining elimination of MIP.

## Materials and methods

**Fig 1:**
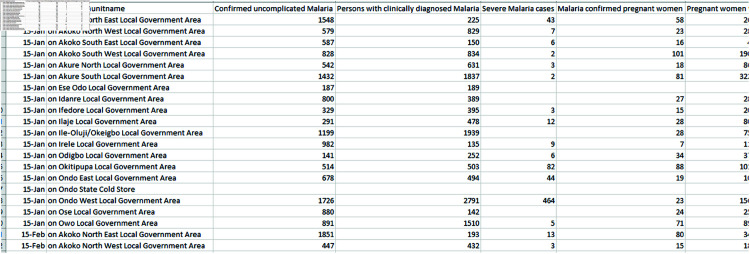
Sample of Routine Dataset for LGA of a State from NMDR.

**Fig 2:**
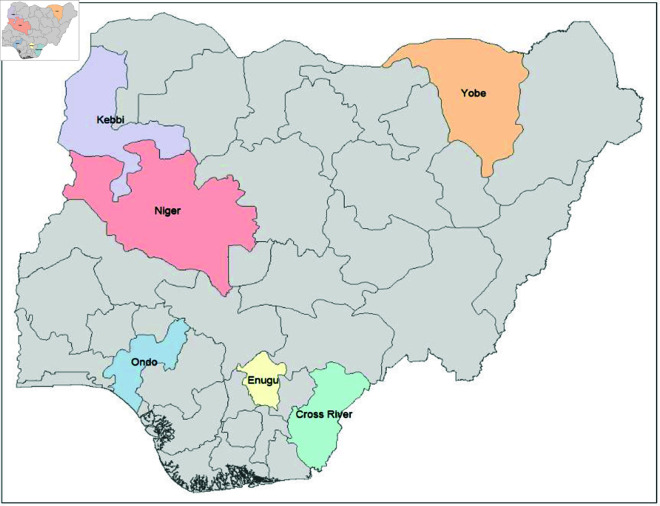
Map of Nigeria with selected States to be studied. Source: Source: Figure generated by the author using shapefiles and geospatial data with R programming, based on country boundary data from GADM, freely available for academic use (https://gadm.org/license.html).

**Fig 3:**
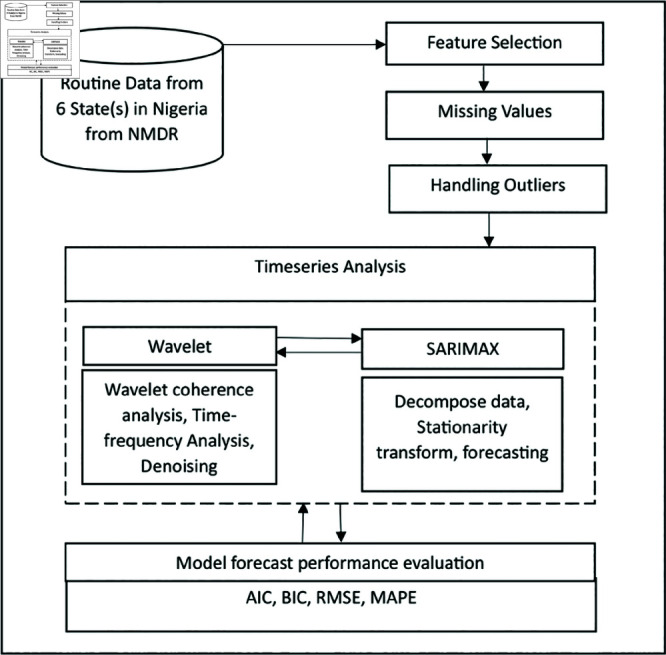
Flowchart for Time series analysis.

The time series analysis for studying the trend of malaria transmission using ARIMA and wavelet involved pre-analysis of Routine MIP cases and the general malaria cases count data (described in Fig 1, from National Malaria Data Repository (NMDR) of NMEP [65], Nigeria for the 6 selected states (shown in Table 1). The monthly Malaria confirmed Pregnant Women and Confirmed uncomplicated malaria cases from routine data of the National Malaria Data Repository was used to carryout the time series for each state or local government area. The complete raw datasets, including the monthly MIP and MC time series for each state or local government area, are available upon request [65]. These States represents high burden State in those 6 geo-political regions in Nigeria as shown in Fig 2. The figure was created by the author using R, based on openly available country boundary data (Administrative boundary data) in figure generation, obtained from GADM [66] (https://gadm.org/license.html), which permits academic use and no copyrighted content was used. Other variables present in the dataset include: Malaria diagnosis status, Case management and interventions, LLIN data, etc. The workflow of the timeseries analysis (as sampled in Fig 3) includes: firstly the pre-analysis and visualization of MIP and MC long-term trend. Secondly, the Variability of MIP incidence over time compared to general MC incidence, which involved analysis of MIP incidence over time compared to that of the general MC incidence was carried out by: i) analysis of MIP incidence seasons of the year, compared to that of MC and ii) Cross-correlation analysis of MIP and MC change over time. Lastly, forecast of short term trends of MIP incidence is generated, based on selected ARIMA or SARIMAX model. The data analysis was carried out using the R Statistical Programming Software. Author-generated code has been made available in the link: https://github.com/oniyelu/Timeseries.git.

**Table 1:** Sampled feature description.

Column code	Feature Name
A	periodname
B	organisationunitname
C	Confirmed.uncomplicated. Malaria
D	Persons with clinically diagnosed Malaria
E	Severe Malaria incidence seen
F	Malaria confirmed pregnant women
G	Pregnant women with clinical malaria

This is a brief description of some of the features in the sampled dataset in Fig 1, used for the time series analysis.

### Pre-analysis and visualization of MIP and MC long-term trend

The confirmed uncomplicated malaria and malaria confirmed pregnant women count data, each representing the MC cases and MIP cases were the data feature selected for time series analysis. These features were preprocessed by carrying out basic statistics to understand the nature of the distribution of the malaria cases data values, to minimize the presence and effect of outliers and to resolve issues associated with missing values. In handling missing values which were observed from a first glance at the data, Shapiro-Wilk Test was carried out on the time series to determine if it is normally distributed, in guiding arrival at the proper value imputation process to adopt. Square root transformation was used to help linearize the data and manage the effects of outliers/handle scaling by stabilizing the variance in the data, which are very helpful for data transformation and regression models [67]. A basic statistics to generate the minimum value, maximum and average mean was also carried out to understand the variance in the data distribution.

Square root transformation ensured scaling transformation keep important patterns and correlations in the data preserved while avoiding overly smoothing. it also helped in reducing skewness and making data more normally distributed. The malaria cases data was made up of discrete counts of MIP and MC cases. Missing data (as summarized in Table 2) were resolved by applying the Kalman smoothen imputation of data for each LGA per year to fill up the missing data of each state. This approach to filling missing values is a robust way to carryout smoothen of the values and account for state-space model that takes into consideration spatial correlations, seasonal effects, and temporal dependencies when imputing missing values. The algorithm followed to carryout the Kalman smoothing is shown as follows:

**Table 2:** Data description and accessing of missing data.

State	Months	LGA	Data points	MC NAs	MIP NAs
Cross Rivers	121	19	2299	129	1067
Enugu	121	18	2178	334	1182
Ondo	121	19	2299	121	1003
Kebbi	121	22	2662	182	1250
Niger	121	26	3146	167	1606
Yobe	121	18	2178	214	1153

1. By estimating the state *x*_*t*_ of a system at time *t*, the Kalman filter operates using: State Equation (Transition Model)
  $$x_{t}=Ax_{t-1}+Bu_{t}+w_{t}$$ (1)
  where: - *A*: models temporal dependencies (such as trends), - *Bu*_*t*_: represents external inputs (e.g., seasonal effects), - *w*_*t*_: process noise (assumed Gaussian).
2. Observation Equation (Measurement Model) is given as:
  $$y_{t}=Cx_{t}+v_{t}$$ (2)
  where: - *y*_*t*_: observed data ( COVID ’19 pandemic effects), - *C*: an observation matrix, - $v_{t}$: measurement noise.
3. The function “build_model()” defines a dynamic process variance (*dW*), meaning *dW* changes according to the impacts of disturbance rather than being fixed. An pandemic-induced disruptions values which occurs between Mar. 2020 - Dec. 2020, are also modeled using exponential decay:
  $$dW=e^{-0.1(t-t_{0})}$$ (3)
  With the assumption that: gradually diminish over time form Jan. 2021 till date. Where: dlm Filter applies Kalman filtering, estimating missing values by recursively updating the state estimate; and dlmSmooth refines predictions by adjusting errors based on historical trends.

The data size of all datapoints was maintained, which was essential for time series, and so records having missing values were not discarded. In analyzing data to better understand the trends, more data points were needed for improved data analysis in AI. Thus the monthly data records for each State were used at the LGA level of malaria cases. Stationarity tests were conducted using the Augmented Dickey-Fuller (ADF) Test [68], which verified if the time series were stationary [68], being an important prerequisite for time series data pre-analysis. The original data and the preprocessed data were both visualized to comparatively understand the need for preprocessing in improving the analysis outcomes. After these preprocessing steps, the plot was further decomposed to understand the long term trend in MIP and MC cases over the years.

### Variability of MIP cases over time compared to general MC cases

Understanding the variability of MIP cases over time compared to that of the general MC cases was approached by i) analysis of MIP cases seasons of the year, compared to that of MC and ii) Cross-correlation analysis of MIP and MC change over time. The trend of MIP and MC over the years happened across the different seasons of the year, thus using the time series plotted according to the seasons of the year, this study looked at the the seasons in which MIP and MC had the highest peaks and related this to the seasons of the year. This showed us at first glance if they both attained their highest peak at the same or close to same time and if the season of the year had any role to play in the outcomes.

Next, the Bi-wavelet coherence analysis [69] was used to examine the cross-correlation in the time series data between MC and MIP cases over the years. Analyses of the pattern changes over time of the MIP cases in relation to changes observed in general MC cases over time. This helped in the understanding of the correlation in the pattern of malaria cases within the pregnant women population, compared to that of the general population in the time and frequency domains. With the aid of the biwavelet analysis being visualised, heatmaps in time and frequency domains, the common behaviour and time-localized patterns was found between the two time series for the various states. The understanding of the data for the frequency content variation over time, are made easier with its ability to quantify the correlation between two time series in the time-frequency domain [70].

### Forecast of future trends of MIP cases

To develop the best ARIMA and SARIMAX model used in forecasting of MIP cases for each sub region, wavelet analysis using the Morlet wavelet transform was first used to carryout the denoising. After denoising, the original and the reconstructed data (denoised data) was visualized to see the output, in comparison to the original data not yet denoised, before carrying out further analysis. The denoised data was then used to carry out parameter estimation for determining the initial parameter values for the ARIMA model of which was further processed with the original MIP data to arrive at the best ARIMA and SARIMAX model for each region. Forecast of a 2 years future MIP cases was generated, based on best fitted forecast after evaluation analyses.

#### Wavelet time series analysis.

Wavelet techniques come in a variety of forms, each with special characteristics that enable them to be applied to various time series analysis applications [71]. A mathematical method known as the Fourier Transform (FT) breaks down a function into a sum of sine and cosine functions (waves) by breaking down a time-domain signal into its component frequencies [72]. When examining the frequency content of signals, this is helpful. An alternative to the FT that offers both time and frequency information is the Wavelet Transform (WT), which breaks down a signal into wavelets, which are confined in frequency and duration [73]. Among the common wavelet techniques are the following: the simplest wavelet is the Haar Wavelet, which looks like a step function [74]. Daubechies created the Daubechies Wavelets, which are employed considerably in signal processing, particularly for noise reduction and data compression, because of its compact support and orthonormal shape [49]. A Gaussian window is utilized by the Morlet Wavelet, a type of wavelet that is often used in time-frequency analysis and offers a good balance between time and frequency localization [49]. Mexican Hat Wavelet, on the other hand, is a Gaussian second derivative, which is the Gaussian function’s negative normalized second derivative [49]. The Morlet wavelet is excellent for time-frequency analysis which was adopted for the analysis in this study. The algorithm followed in analysing the data involves the following:

1. Define and set up Parameters (a change in malaria cases values , number of scales (i) that can be changed as needed, and angular frequency). Next, the scale-adjusted Morlet wavelet function is defined. Scale-adjustable Morlet Wavelet Function ana algorithm is given as:
  $$\psi (t,s)=e^{i\cdot 2\pi t/s}\cdot e^{-t^{2}/(2s^{2})}$$ (4)
  where *t* is time, and *s* is the scale.
2. Next, make scale and loop adjustments to increment *j* by 2 Until j≤n:
  $$s_{j}=1,3,5,\ldots ,n$$ (5)
  where *s*_*j*_ represents the scales.
3. Initialize Wavelet Coefficients Matrix:
  $$W_{j,k}=0$$ (6)
  for all *j* and *k*, where *W* is the wavelet coefficients matrix.
4. Use Adjusted Scales to Calculate Wavelet Coefficients:
  $$W_{j,k}=\sum_{t}x(t)\cdot \psi (t-k,s_{j})$$ (7)
  where *x*(*t*) is the signal.
5. Compute Wavelet Power Spectrum:
  $$P_{j,k}=|W_{j,k}{|}^{2}$$ (8)
6. Convert Scale to Fourier Period:
  $$T_{j}=\frac{1}{s_{j}}$$ (9)
7. Take the real part of the matrix coefficient and get a denoised time series back together:
  $$\hat{x}(t)=\text{Re}\left(\frac{1}{J}\sum_{j=1}^{J}W_{j,t}\right)$$ (10)
  where $\hat{x}(t)$ is the denoised time series.

#### ARIMA model for time series analysis and forecasting.

For forecasting to be effective, non-stationary data must be converted into stationary data. This was done by using differencing which is a component of ARIMA’s “Integrated” component of the ARIMA and SARIMAX model applied to the time series. Once the series was stationary, the training of the ARIMA model was carried out. This involved specifying the order of the autoregressive (AR), differencing (I), and moving average (MA) components. The residual check on the models are used to show if the models could not fully capturing the variance structure (i.e., conditional heteroskedasticity), or the serial correlation in the data. If present, this could limit the reliability of the forecasts generated and violate key ARIMA assumptions (white noise residuals). To account for the seasonality present in the data and other exogenous factors, SARIMAX model was also adopted to carryout comparism on both model in analysing the data. Exploring Seasonal ARIMA (SARIMAX), for autocorrelation and heteroskedasticity that could be due to seasonalilty or other factors that ARIMA failed to capture, are further analysed using seasonal differencing and seasonal AR/MA terms.

The SARIMAX (Seasonal AutoRegressive Integrated Moving Average with Exogenous Variables) model which extends SARIMAX by incorporating external factors (exogenous regressors) was analysed. COVID ’19 pandemic disruptions deduced from the data was one of the exogenous factors considered and eventually used. IPTp coverage data was another exogenous factor initially considered, but it was observed that the IPTp data from DHIS was a yearly point data. Although the State level IPTp data was available, however aggregating MIP cases data to get the it as yearly data to maintain the same scale before carrying out forecast was however not appropriate. Also, LGAs level IPTp data for each State coverage level were not accessible, thus IPTp as an exogenous factor was not used. Further analysis and decision-making was established by utilizing wavelet analysis in combination with SARIMAX to improve forecasting and analysis of the underlying patterns and fitness of model to the time series data.

The reconstructed wavelet time series: $\hat{x}(t)$ was used by the algorithm within the given model: *ARIMA*(*p*, *d*, *q*). The Objective function AIC was used repeatedly to identify the optimal model: Minimize AIC, and finally generating forecasts: $\hat{x}(t\;+\;h)$ with 95% confidence intervals. Once the optimal ARIMAX and SARIMAX model fit had been obtained, the model’s coefficients were utilized as starting points for model optimization through the application of the Kalman filter [48]. The ranking criteria was ranked by prediction error measures (including: Mean Absolute Percentage Error (MAPE), Root Mean Squared Error (RMSE).

The algorithm to carryout the ARIMA modelling approach follows thus:

1. After the reconstructed the wavelet time series is generated: $\hat{x}(t)$, SARIMAX models which extends ARIMA is defined by adding:
  - Autoregressive (AR) Component
    $$\phi (B)y_{t}=\theta (B)\varepsilon_{t}$$ (11)
    where ϕ(B): AR terms, θ(B): MA terms, and $\varepsilon_{t}$: white noise.
  - Integration (I) Component: To ensure stationarity, differencing is carried out
    $$\nabla^{d}y_{t}=(1-B{)}^{d}y_{t}$$ (12)
    where *B*: backshift operator.
  - Moving Average (MA) Component
    $$y_{t}=\mu +\sum_{i=1}^{q}\theta_{i}\varepsilon_{t-i}+\varepsilon_{t}$$ (13)
  - Seasonal ARIMA (SARIMAX) Extension captures seasonal patterns with additional terms:
    $$\Phi (B_{s})y_{t}=\Theta (B_{s})\varepsilon_{t}$$ (14)
    where *B*_*s*_ is seasonal backshift and (*P*, *D*, *Q*) defines seasonal behavior.
  - Exogenous Variables *X* in which SARIMAX incorporates external predictors:
    $$y_{t}=\beta X_{t}+\phi (B)y_{t}+\theta (B)\varepsilon_{t}$$ (15)
    where $\beta X_{t}$ adjusts for external factors (pandemic disruptions based on data).
    Note that: Model selection was done using AIC (Akaike Information Criterion). Training/test split of dataset was based on a 70% train set in training the models and 30% test set applied in carrying out evaluation of model parameters performance). Exogenous effects (train_disruption, test_disruption) are applied to modify predictions. Performance evaluation was done using RMSE and MAPE
2. The optimal SARIMAX model was chosen using the Akaike Information Criterion (AIC):
  AIC=2k−2ln(L) (16)
  where: - *k*: the number of parameters in the SARIMAX model. - *L*: the likelihood function of the model.
  - Iteration over different combinations of parameters (p, d, q) and (P, D, Q) is done while tracking AIC values to determine the best model. To find the best model, there is a loops over possible values of *p*, *d*, and *q*, as well as their seasonal counterparts *P*, *D*, and *Q*:
    for p=0 to P (17)
    for d=0 to D (18)
    for q=0 to Q (19)
    With the addition of external regressors and seasonal components, each iteration fits an ARIMA model.
3. By include exogenous regressors, the generic SARIMAX formulation expands upon ARIMA. *X*_*t*_, which given as:
  $$\hat{x}(t)=\phi_{1}\hat{x}(t-1)+\phi_{2}\hat{x}(t-2)+\ldots +\phi_{p}\hat{x}(t-p)+$$ (20)
  $$\epsilon (t)+\theta_{1}\epsilon (t-1)+\theta_{2}\epsilon (t-2)+\ldots +\theta_{q}\epsilon (t-q)+\beta X_{t}$$ (21)
  where: - $\phi_{i}$: the autoregressive coefficients. - $\theta_{i}$: the moving average coefficients. - ϵ(t):the error term. - $\beta X_{t}$: captures the effect of the exogenous variable (i.e pandemic disruption).
4. After fitting each possible SARIMAX model, the AIC values are compared:
  $${\text{AIC}}_{p,d,q}=2k_{p,d,q}-2\ln(L_{p,d,q})$$ (22)
  The best model is selected as:
  $$\text{Best Model}=\arg\min_{p,d,q}{\text{AIC}}_{p,d,q}$$ (23)
5. Forecasts with 95% Confidence Intervals was created by using the optimal ARIMA model. $\hat{x}(t+h)$ for *h* steps ahead:
  $$\hat{x}(t+h)=\phi_{1}\hat{x}(t+h-1)+\phi_{2}\hat{x}(t+h-2)+\ldots +\phi_{p}\hat{x}(t+h-p)+\epsilon (t+h)$$ (24)
  $$+\theta_{1}\epsilon (t+h-1)+\theta_{2}\epsilon (t+h-2)+\ldots +\theta_{q}\epsilon (t+h-q)$$ (25)
  The forecast’s 95% confidence interval is given by:
  $$\hat{x}(t+h)\pm 1.96\cdot \sigma_{\hat{x}(t+h)}$$ (26)
  where $\sigma_{\hat{x}(t+h)}$ is the forecast’s standard error.

To include RMSE (Root Mean Squared Error), MAPE (Mean Absolute Percentage Error), and ACF (Autocorrelation Function) in the evaluation of the best ARIMA model, AIC balanced model fit and complexity, RMSE measured the average magnitude of the prediction errors and MAPE measured the prediction accuracy as a percentage. These loss functions helped in evaluating and selecting the best model for the data. The function to calculate these metrics for each model were included in defining the function to fit the ARIMA model based on AIC. After getting the best fit ARIMA model, the AC,F PACF,and the test of the residual error including Jarque-Bera (JB) Test for Normality, Lagrange Multiplier (LM) Test for ARCH Effects and Ljung-Box Q (LB-Q) Test for Autocorrelation [75] was also carried out to evaluate the goodness of the selected models fit to the data. The definition of the metrics are as follows:

1. The AIC is a measure of the relative quality of a statistical model for a given set of data. It balances the goodness of fit of the model with the complexity of the model.
  Mathematically, AIC is defined as:
  AIC=2k−2lnL (27)
  where: - *k* is the number of parameters in the model. - *L* is the maximum value of the likelihood function for the model.
2. Root Mean Squared Error (RMSE) is a measure of the differences between predicted values and observed values. It is the square root of the average of the squared differences between predicted and observed values.
  Mathematically, RMSE is defined as:
  $$\text{RMSE}=\sqrt{\frac{1}{n}\sum_{i=1}^{n}(y_{i}-{\hat{y}}_{i}{)}^{2}}$$ (28)
  where: - *y*_*i*_ is the observed value. - ${\hat{y}}_{i}$ is the predicted value. - *n* is the number of observations.
3. Mean Absolute Percentage Error (MAPE) is a measure of prediction accuracy of a forecasting method. It expresses the error as a percentage of the actual values.
  Mathematically, MAPE is defined as:
  $$\text{MAPE}=\frac{1}{n}\sum_{i=1}^{n}\left|\frac{y_{i}-{\hat{y}}_{i}}{y_{i}}\right|\times 100\%$$ (29)
  where: - *y*_*i*_ is the observed value. - ${\hat{y}}_{i}$ is the predicted value. - *n* is the number of observations.
4. Jarque-Bera (JB) Test for normality checks if the p-value of model was > significance level (p-value *geq* 0.05), then the residuals were normally distributed.
5. Lagrange Multiplier (LM) Test for ARCH Effects checks if the p-value was greater than significance level, then there were no significant ARCH effects present.
6. Ljung-Box Q (LB-Q) Test for autocorrelation checks if the p-value was less than your chosen significance level, then there were significant autocorrelation in the residuals.

## Results and discussion

### Analysis of long term trend in MIP cases

**Fig 4:**
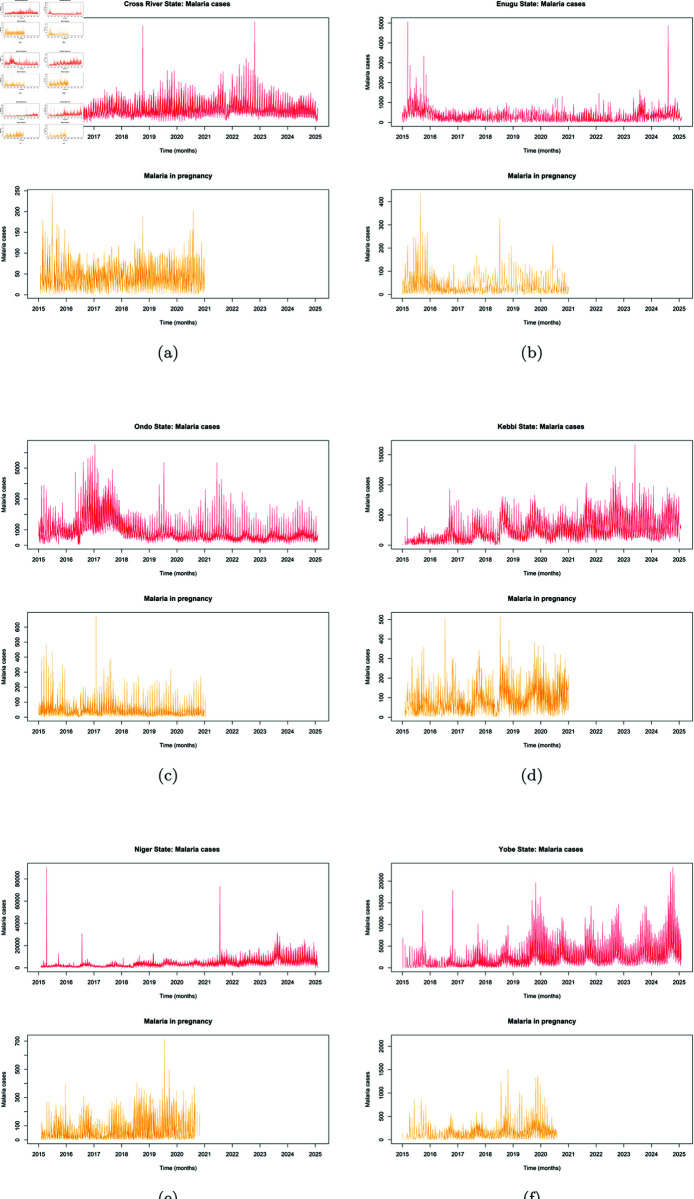
Raw data Time Series visualized for Counts of Malaria cases and Malaria in Pregnancy cases.

**Fig 5:**
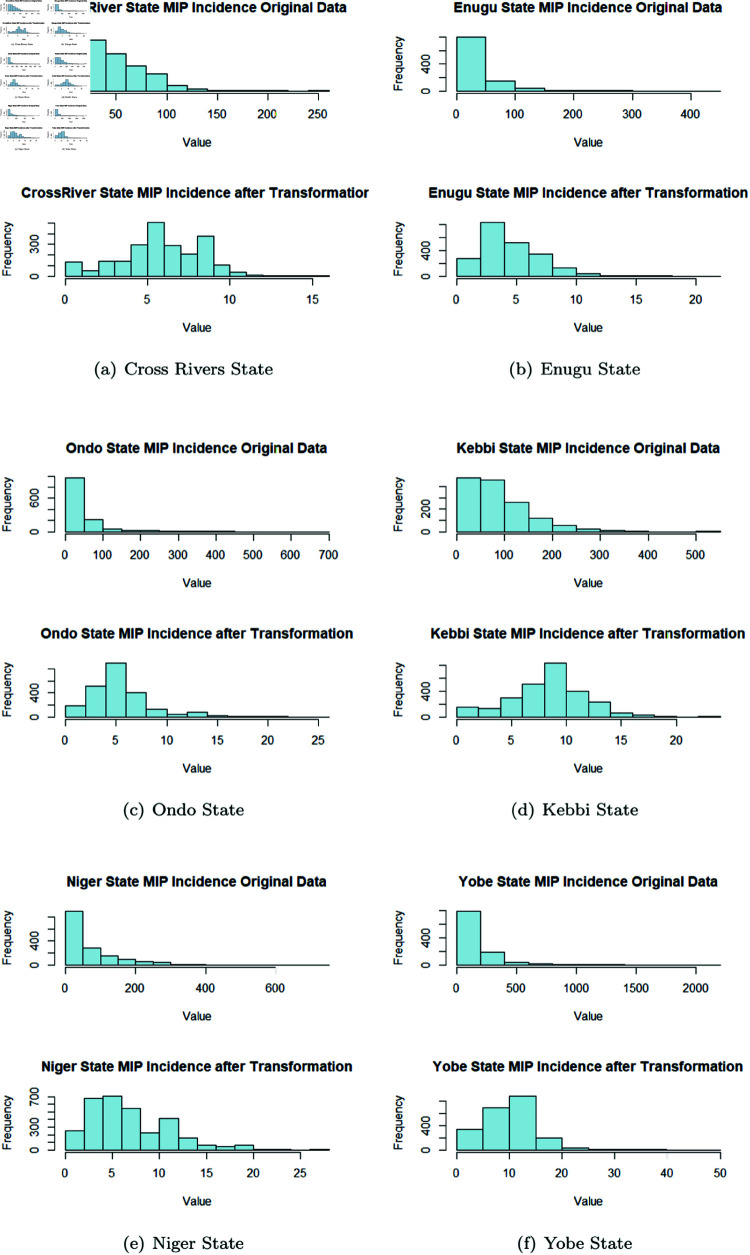
Histogram Distribution of each State’s data before and after applying Square root transformation.

As noted by [76], when data is not preprocessed, it can affect the eXplainability of methods used in analysis and the overall integrity of results generated after analysis. Preprocessing of data is very essential and the importance of more data points is needed to enhance the accuracy and applicability of model analysis as observed in [77]. To really understand the long term trend in MIP cases, a closer look at the time series was done by firstly visualizing its original time series state. As seen in the time series plots Fig 4, it was observed that there exist several missing data in the MIP time series, compared to the MC time series plot. The missing record were based on no updates to records of MIP majorly from year January 2021 to January 2025. To help determine the values for imputing in replacing all missing values, each time series data for each State was further tested to determine if it had a normal distribution, by applying Shapiro-Wilk normality test (results as shown in Table 3) and plotting the histogram (as shown in Fig 5) of the MIP.

**Table 3:** Summary of the Shapiro-Wilk normality test for the 6 regions of Nigeria.

Region	State in view	w	p-value	histogram
South south	Cross Rivers	0.9202	2.2e-16	Right skewed
South East	Enugu	0.6824	2.2e-16	Right skewed
South West	Ondo	0.6141	2.2e-16	Right skewed
North West	Kebbi	0.8928	2.2e-16	Right skewed
North Central	Niger	0.7817	2.2e-16	Right skewed
North East	Yobe	0.6588	2.2e-16	Right skewed

It was observed from the Shapiro-Wilk test, that all the timeseries were not normally distributed as shown in the histogram plot which showed most of them to be heavily right skewed. To confirmed this, although the w values were close to one, their significance levels were all low because the various p-values of the Shapiro-Wilk test were lower than 0.05. To directly use appropriate value rather than just a mean value to replace missing data, Kalman smoothing imputation was used to account for temporal dependencies, seasonal effects, or spatial correlations. All of these are important for the malaria data analysis to each of the time series, which are State specific and recorded according to the LGAs per year. Square root transformation was applied before the Kalman imputation to replace missing values, which reduced the level of skewness and still preserved the integrity of the data patterns as much as possible. This was based on the preliminary statistical test carried out to check for the overall mean of the time series, the median, minimum value and maximum values. It was observed that the median and mean of Cross rivers State and Kebbi states was greatly differed from the maximum value. This called for closer observation of the time series of these State and not surprisingly, a single distinct value of 110022 and 2921 was observed in the MIP data of Cross Rivers State and Kebbi State. These were extremely huge, compared to the next largest values in Cross Rivers State and Kebbi State times series respectively, which was just in its 2 hundreds in unit. Thus this outliers where edited to be represented as 22 and 292 respectively. To avoid tampering with the data, only these two very obvious outlier points where edited before the application of the square root transform to the data.

**Fig 6:**
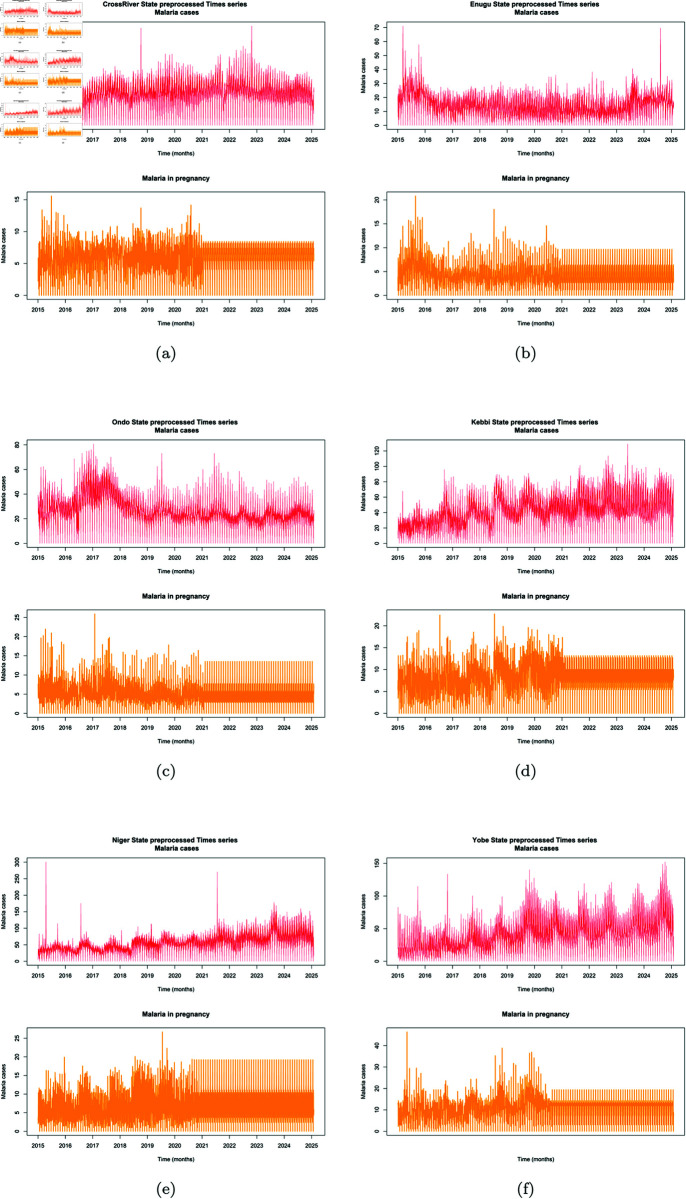
Preprocessed time series.

The preprocessed time series was plotted as seen in Fig 6). Although values for the missing years (2021–2024) in the MIP data was generated for each LGA per year, before forecast, the decomposition of the MIP data and other analysis was limited to December 2020 of original available MIP data. Forecast of MIP records for the 2 years 2025 & 2027 were generated as two year points ahead are extrapolated forecast based on older data within the range between 2015 to 2020 records. Due to the lack of post-2020 inputs, the forecast results are thus advised to be interpreted with caution From the plotted original time series and the plotted preprocessed time series, it is obvious that the trends of the time series are preserved and missing values filled up with reasonable Kalman smoothened estimates. The range of the variance in the preprocessed data is thus better managed, to arrive at a good analysis in the end.

**Fig 7:**
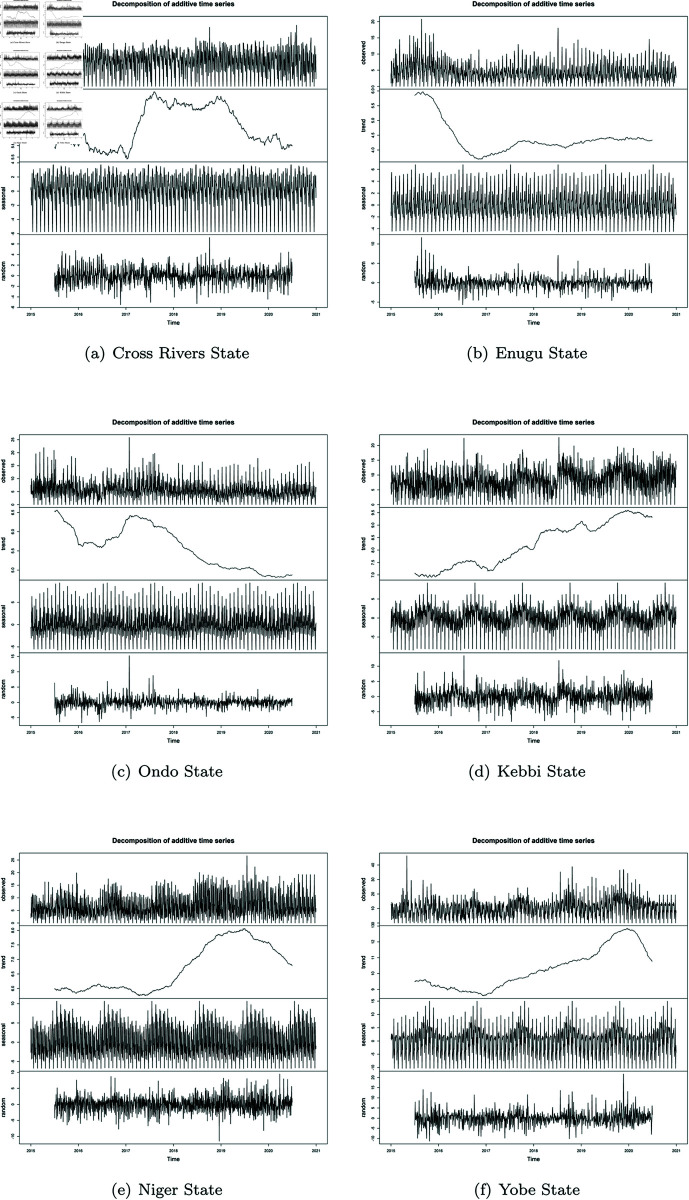
Decomposed time series.

The time series plots were further decomposed as shown in Fig 7), to understand the long term trend in MIP cases over the years form the available data from January 2015–December 2020, separating inherent random noise in the observed time series data. At first glance of the preprocessed time series plot, one would say that the the trend in the general MC and MIP plot seem to follow similar pattern. The shape of the patterns seem not too different in the various States, with just some little variations in the fluctuating growth rate of cases per state. However, decomposing the data, showed clearly the huge variability inherent. From the plot, it could be seen that Cross Rivers State trend assumed a little peak at 5.7 in mid-2015, and then a downward turn to a trough towards the end of the first quarter of 2016. This low growth was characterized by some fluctuations of moving average around 5.6 up until the second quarter of 2017. A sharp trough below 5.6 was observed and immediately followed by a sharp peak reaching 6.2 within the first and third quarter of year 2017. Some slight troughs and fluctuations between the 4th quarter of 2017 to the end of third quarter of 2019. A steep trough was then seen which continued to steep till 2020. The MIP cases in Cross Rivers State showed the random noise mostly around -4 and 4 change in cases, with a sharp outlier at cases 8 in towards last quarter 2018.

Enugu State on the other hand started on a high peak in mid-2015, but a steep trough in 2016 and the lowest point attained within the fourth quarter of about 3.6 change in cases, which was sustained until the beginning of 2017. There was a minimum rise to peak of 4.5 within the third quarter of 2017 and fluctuating down back to a trough of about 4.3 again around the third quarter of 2018. This minimum fluctuation was sustained to the end of 2020. The MIP time series on the other hand had the random noise ranging between -3 and 3 through the years with some spikes of to 12 in third quarter of 2015 and around 5 cases in the mid of 2018. It was observed in Ondo State that the actual trend showed relatively steeps slope to a trough from mid-2015 in cases from 6.6, down to 5.6 by the beginning of 2016. This was sustained with minimal fluctuations up until end of 2016, where a rise in the fluctuations of additive growth going up to a peak of 6.5 by the first quarter of 2017. Before the end of this quarter, the trend began a downward trend through the years and reaching a minimum trough of about 5.0 by the end of 2020. The random noise within the MIP data ranged roughly within -5 and 5; with an distinct as high as 15 in cases in 2017.

The trend of MIP in Kebbi State started from a trough at moving average of 7.0 and gradually rose to 8.0 past midway of 2016, which was immediately followed by a fluctuation downward to a trough at 7.5 by the beginning of 2017. Before the end of the first quarter, a steady rise in the growth trend was resumed which kept rising with some minimal fluctuations to troughs, and then reaching a maximum peak of 9.5 towards the end of 2019. The MIP time series had the random noise ranging between -5 and 5 with two distinct peaks reaching point 14 and 12 at mid-2016 and mid 2018 respectively.

The trend of MIP in Niger State started in 2015 at a trough, rising and falling with little fluctuations between cases moving average 6.0 and 6.4, and moving back to trough of 5.8 by the middle of 2017. A gradual rise in the MIP trend began from this point up until mid 2019, with a record peak of about 8.0. The MIP time series had the random noise ranging between -5 and 5. MIP trend in Yobe State in mid-2015 was at trough of 9.5, with a little peak then back to the lowest trough recorded at in cases moving average of 8.8 by 2017. From this point, steady rise in the trends growth rate was sustained to 2020, reaching the highest peak recorded of 13.0. The MIP time series had the random noise ranging between -5 and 10, with a few spikes up to 20.

It was observed from the results (as shown in Table 4) that while Cross River State experienced its highest peak in MIP cases around 2017 and 2019, Enugu had its low within that same period. Although the North-Central (Niger State) recorded the lowest range in the trend of MIP cases among the Northern regions, ranging between 6.0 and 6.4; however the highest peak of the trends in the South-South, South-East, and South-West (6.5 cases) was observed to be less than the lowest possible trough of even the other North-East and North-West states (9.5 and 7.5 respectively). This indicates the higher prevalence of MIP in the northern regions of the country, which aligns with various reports on malaria prevalence in Nigeria, with the North accounting for most of the high-burden states. Varying presence of seasonal pattern was shown in all the time series data of the 6 states, with regular, repeating patterns in malaria cases that occurred at fixed intervals. With the different seasonal patterns shown by each region, serving as pointers to the fact that there are certain factors that are the major drivers of malaria transmission trend peculiar to each region. Thus to reduce MIP cases, a good under standing of the geographical location/zones and other peculiar exogenous factors occurrence such as socio-economic, seasonal practices and well as climatic and environmental factors , must be understood to achieve a good result. This variability shows the peculiar to their different spatial location, environmental and social practices in the different locations and the fact these play a huge role in the varying malaria transmission patterns observed.

**Table 4:** Summary of long-term trends in 6 regions of Nigeria.

Region	State in view	MIP Trend	MIP Highest peak
South south	Cross Rivers	sharp peak, slight troughs	6.2 (2017 and 2019)
South East	Enugu	Begin High peak but sharp trough	6.0 (2015)
South West	Ondo	Relative downward trend sustained	6.5(2015)
North West	Kebbi	relative fluctuating but increasing trend sustained	9.7 (2019)
North Central	Niger	Significant increasing trend, then downward trend	8.0 (2021)
North East	Yobe	Relative upward trend sustained, then downward trend	13.0 (2017)

### Analysis of variability over time of MIP compared with general MC cases

The analysis of the variability of MIP cases was be approached by: i) analyzing how MIP cases vary with seasons of the year and compared to that of the general MC and ii) analyzing the cross-correlation between MIP and MC patterns over the years.

#### Analysis of malaria in pregnancy cases varying with seasons of the year.

**Fig 8:**
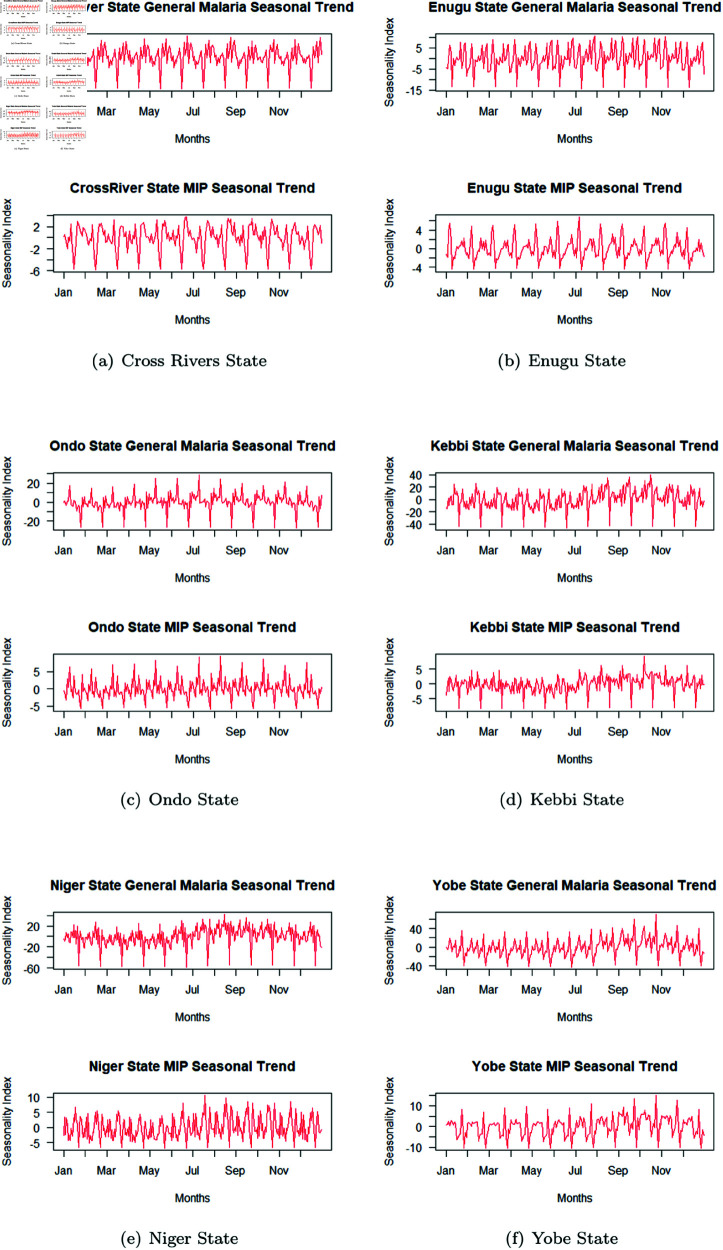
Analysis of malaria in pregnancy cases varying with seasons of the year.

From the time series analysis carried out to observe how MIP vary with the seasons of the year (as shown in Fig 8), it is observed in Cross rivers State that there is a repeated pattern in the change in MIP cases all round the year ranging between -6 and 3, however there were two highest peaks around the 3rd week June & beginning of September. On the other hand, MC cases all year round ranged between -30 and 20, with a peak around 18 in mid June.

Enugu State had a repeated pattern the MIP cases all round the year ranging between -4 and 5 of malaria cases change, with highest peak around 1st week July. The MC cases recorded changes within -15 and 10 with really no obvious peak shown except just slightly at end of July. Ondo State seemed to maintain a repeated pattern in the MIP cases all round the year between -5 to 10 of malaria cases change, with about 4 months having competitive high range of malaria cases and the highest around the 2nd weeks in July and August with a peak of 2. In the case of MC seasonal variation, a repeated pattern was observed all round the year with at peaks and troughs between -25 to 25, with about 4 months having competitive high range of malaria cases and the highest peak in the 2nd week July. Kebbi State MIP cases shows changes ranging between -10 and 5, with highest peak attained towards end of 1st week in October. The MC cases change on the other hand ranges between -40 and 40, with the highest peak attained in mid October.

Niger State showed a repeated pattern in the MIP cases all round the year between -5 to 8 of malaria cases change, with the highest peak around the Mid August at about 10. MC cases change ranged between -60 and 30, with the highest peak at point 10 also in Mid August. Yobe State had its repeated pattern of the MIP cases all round the year between -10 to 8 of malaria cases change, with about 3 months attaining competitive high peaks of malaria cases and the highest around the 4th weeks in October with a peak of 12. On the other hand the MC cases change range between -40 and 40, with the highest peak attaining point 60 in the 4th weeks in October.

Analysis of MIP showed to vary with seasons of the year (as summarized in Table 5), with the peak months for MIP cases recorded were: South-South (3rd week June & beginning of September) MC 3rd week October, South-East (1st week July) MC end of July, South-West (2nd weeks in July and August) MC 2nd week July, North-West (1st week October) MC mid October, North Central (Mid August) MC Mid August and North-East (3rd weeks in September and October) MC 3rd week October, respectively. It can be noted that South-south, South-West and North-East had their highest seasonal MIP peaks twice in a year, MIP cases in all regions experienced most of their peak MIP seasons before MC, except for North -central which had both MIP and MC peak season occur approximately same week (Mid August). These are pointers to the fact that to reduce MIP cases, a good understanding of the geographical location/zones and other peculiar seasonal occurrence such as socio-cultural seasonal practices and well as climatic and environmental factors, must be understood to achieve a good result. Also, intervention for MIP must be strategic, and specific to the pregnant women population, who in most cases experience their peak cases before the general MC cases. This variability shows the peculiar to their different spatial location, environmental and social practices in the different locations and the fact these play a huge role in the varying malaria transmission patterns observed.

**Table 5:** Summary of analysis of MIP and MC cases change with seasons of the year.

Region	MIP peak	MC Peak
South south	3rd week June & Beginning of September	mid June
South East	1st week July	End of July
South West	2nd weeks in July and August	2nd week July
North West	1st week October	Mid October
North Central	Mid August	Mid August
North East	4th weeks in October	4th week October

#### Cross-correlation analysis of MIP and MC change over time.

**Fig 9:**
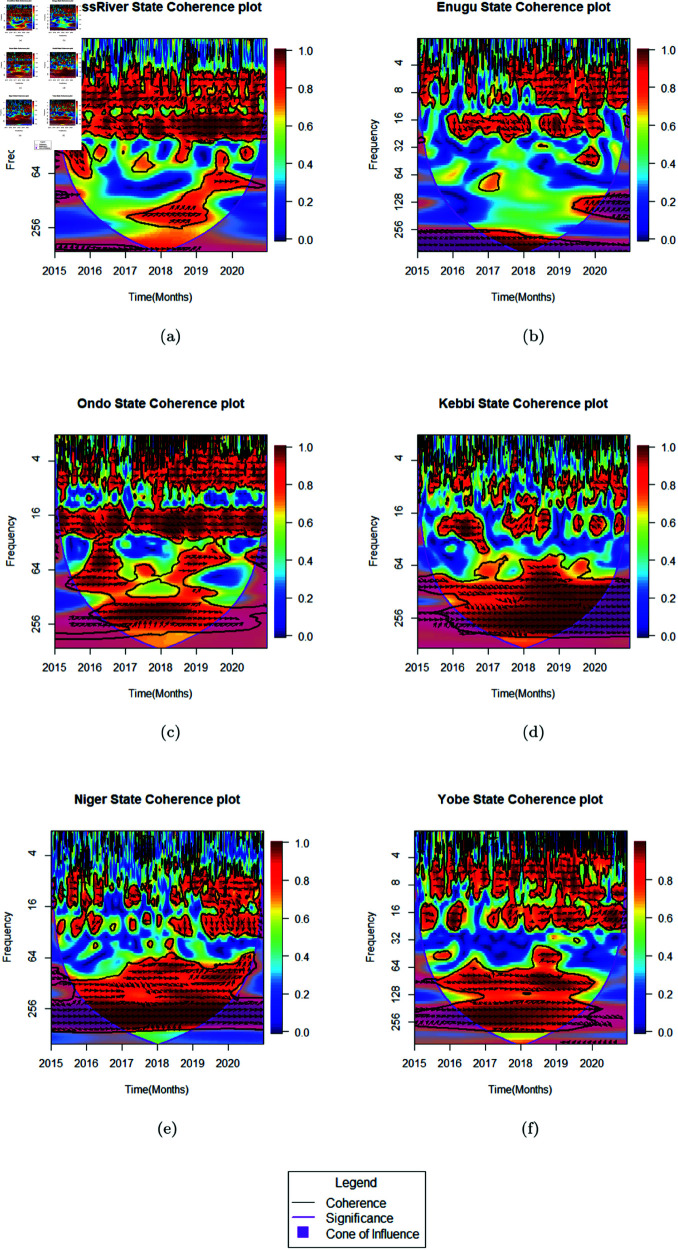
Change over time of MIP compared with general MC cases.

The analysis of the change over time of MIP compared with the general MC cases, was done using Bi-Wavelet coherence (as shown in Fig 9, to Understand the correlation in occurrence of peaks of malaria cases in the 6 regions of Nigeria as the years go by. For the Bi-wavelet analysis of cross- correlation between MIP and MC cases over the years, the historical data between 2015–2020 is used due to the missing updated records on MIP cases. Instead of interpreting frequency directly in Hertz, the coherence cycles are translated into real-world malaria transmission periodicities [78,79]. Lower frequency bands (e.g., 8–32 Hz) reflect seasonal malaria trends, while higher frequencies (e.g., 128–256 Hz) capture short-term fluctuations driven by localized peaks or intervention effectiveness. In support with [81] the coherence between MIP and MC at these frequencies indicates synchronized malaria transmission patterns, of which MIP was seen to often preceding MC peaks.

In Cross River State, the coherence analysis indicated weak malaria endemicity, with low-frequency cycles (approximately 2–4 months) showing patchy but weak coherence throughout the year. However, intermittent coherence was observed between 2015–2020, particularly in short-term cycles (1–2 months) and seasonal variations (approximately 6–12 months) during specific years such as 2016, 2020, mid-2015 -2016, and 2018–2019. This suggested a partial relationship between MIP and MC, but their transmission peaks did not strongly synchronize. This implied that exogenous factors were likely influencing malaria trends. MIP led MC in transmission periods, implying a consistent yet indirect relationship. MIP cases could thus be early indicators of broader malaria transmission fluctuations. The high rainfall and strong river currents in Cross River State likely contributed to reducing mosquito breeding sites, which could limit malaria transmission compared to some other regions. Also, higher education levels based on civilization population among women in the region and thus strong adherence to IPTp may have helped sustain the State’s lower malaria burden.

Enugu State coherence spectrum showed strong periodic relationships at lower frequencies (approximately 6–8 months) persisting throughout the year with patches of coherence from 2015–2020. A short-term transmission cycle (2 months) observed in 2017 could be a pointer to localized synchronization between MIP and MC, but this pattern did not persist across all years. MIP seemed to precede MC transmission cycles, reinforcing a connection between pregnancy-related cases and broader malaria transmission patterns. Peaks were not fully synchronized, suggesting intervention timing and environmental factors could play a role. Enugu’s higher urbanization levels, education, and possibly strong preventive health measures could have helped in keeping malaria endemicity relatively low compared to northern States.

For Ondo State, the coherence analysis showed moderate malaria endemicity, with sustained high-frequency cycles (approximately 2–4 months) from mid-2016 to 2019. Additional coherence appeared at longer cycles (approximately 6–12 months) between 2016 and 2017, suggesting seasonal alignment between MIP and MC transmission trends. Urbanization, improved healthcare access, and higher education exposure may have contributed to malaria not being as severe compared to some other endemic regions.

Kebbi State showed strong malaria endemicity, with coherence observed across rapid malaria transmission cycles (approximately 1–3 months) from 2015 to 2020. Additional coherence at longer cycles (approximately 6 months) between 2016 and 2017 suggested seasonal variations in transmission. MIP consistently led MC, reinforcing pregnancy-related susceptibility in malaria transmission. However, peaks do not align, meaning other exogenous factors such as environmental or socioeconomic factors (IPTp adherence, healthcare access) influence the malaria trends. Socioeconomic disparities of women whose level of literacy and economic power of the majority may be below average, strong seasonal effects, and IPTp adherence could have contributed to higher endemicity in this region.

Malaria transmission in Niger State showed strong endemicity, with broad coherence observed across high-frequency cycles (approximately 2–4 months) from 2015–2020, including patches around longer seasonal cycles (approximately 6 months). MIP largely preceded MC transmission peaks, reinforcing the potential for MIP cases to serve as a leading predictor for malaria peaks. Peaks did not fully overlap, likely due to external intervention effects (IPTp adherence, rainfall patterns, socioeconomic access to treatment).

Yobe State showed significant malaria endemicity, with coherence observed in fast transmission cycles (approximately 2–3 months) between 2016–2019, and additional long-term trends (approximately 6 months) scattered throughout. MIP preceded MC cycles, confirming a consistent but non-synchronized relationship between pregnancy-related malaria and general malaria transmission. The strong malaria burden in Yobe likely resulted from socioeconomic disparities of women whose level of literacy and economic power of the majority is below average. Healthcare accessibility issues, and seasonal mosquito breeding patterns also play a role in this State. Coherence strength at different frequencies reflects how malaria in pregnancy (MIP) and malaria cases (MC) are synchronized across transmission cycles.

It should be noted that while States with higher numbers of LGAs (e.g., Niger: 26 LGAs, Kebbi: 22 LGAs) could have a denser dataset, this did not necessarily mean increased detection of high-frequency malaria trends. Rather, greater LGA coverage provided a more statistically robust representation of malaria incidence patterns across local populations. The presence of strong coherence at specific frequencies indicated alignment in MIP and MC transmission cycles, suggesting shared environmental or epidemiological drivers of malaria incidence. Seasonal trends, intervention timing, and fluctuations in malaria transmission played a more significant role in shaping coherence. After analyzing malaria trends across regions, the strongest coherence between MIP and MC occurred in short-term transmission cycles (approximately 2–4 months per cycle) in North-West (Kebbi), North-Central (Niger), and North-East (Yobe), followed by South-West (Ondo). Weaker coherence was observed in South-South (Cross River) and South-East (Enugu). This aligns with documented higher malaria prevalence in northern Nigeria, likely driven by lower IPTp adherence, socioeconomic disparities, and favorable environmental conditions for mosquito breeding. The consistent finding that MIP precedes MC transmission cycles suggests pregnancy-related malaria could serve as an early indicator of broader malaria transmission outcomes.

The cross correlation of change over time of MIP compared with the general MC cases in the populations of the different regions is summarized in Table 6), Coherence strength at different frequencies reflects how MIP and MC are synchronized across transmission cycles.

**Table 6:** Summary of cross-correlation analysis of MIP (2015–2020).

Region	Endemicity	Broadest band	Periodicity
South south	weak	16 Hz	Long-term (6–12 months)
South East	weak	8 Hz	Extended (12+ months)
South West	Strong	256 to 128 Hz	Short-term (1–3 months)
North West	Strong	256–64 HZ	Short- mid-term (1–6 months)
North Central	Strong	256 to 32 Hz	Rapid transmission cycles (1–6 months)
North East	Strong	256–128 HZ	High-frequency cycles (1–3 months)

The high endemicity observed in the northern states is in agreement with the reports of high burden of malaria prevalence mostly in the northern states of Nigeria. It is also noticed that for most of the regions, MIP leads MC in peak, which is could be a pointer to the fact that as a vulnerable population, being the population firstly prone to the surge of malaria as the seasons of the year comes and goes. The consistent finding that MIP precedes MC transmission cycles suggests pregnancy-related malaria could serve as an early indicator of broader malaria transmission outcomes.

### Forecast of future trends of MIP cases using an hybrid of Wavelet - ARIMA and SARIMAX models

**Fig 10:**
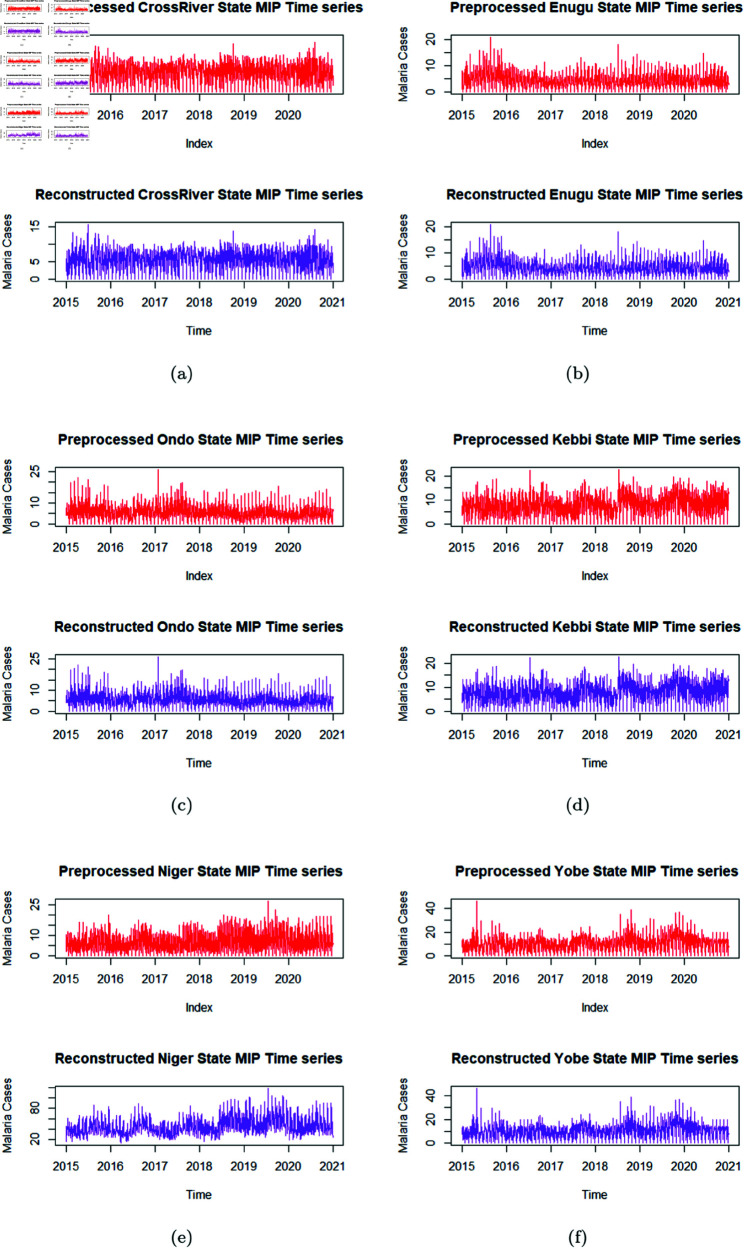
Denoising of the time series data.

Wavelet analysis and denoising of time series for specified change in malaria cases at amplitude (A = 7.99 e-04) and scale (J = 20) was applied after it was adjusted per time using Morlet wavelet. The denoising of the time series data of the original and then reconstructed (denoised data) was obtained as visualized in Fig 10, to optimize modelling process by minimizing noise in data for further analysis. The denoised data was then used to determine the initial ARIMA and SARIMAX model parameter values before the estimation of actual model parameters, analyzed for forecast of future trends. The starting and ending point (p,0,0 and 3,2,*q* respectively) for the analysis ARIMA models parameter estimation where *q* was based on the initial ARIMA parameter values, with *p* = 1 for all initial value. The SARIMAX also took the same format, with the seasonal parameter estimated also having its own start and end parameters (0,0,0 and 2,2,2 respectively).

MIP data from the NMDR basically had no records from 2021 till date, so to fill in the missing values the previously generated Kalman smoothened values used during preprocessing were adopted. In determining the stationarity of the time series for better analysis when using ARIMA or SARIMAX model, all the time series where observed to be stationary after applying the augmented dickey-fuller test. This analysis was done on the granular LGAs level data of the monthly cases per State. The initial SARIMAX parameters where determined based on the denoised time series of each State, to achieve better estimates of the ACF and PACF, in determining the initial AR-I-MA (p,d,q) parameters (as shown in Table 7.

**Table 7:** Parameters of the initial ARIMA model.

State	ADF: log(p-value)	Integral	Initial (p,d,q)	initial AIC
Cross Rivers	-12.978(0.01)	ln(0)	1,0,3	4171.03
Enugu	-6.9828(0.01)	ln(0)	1,0,4	4078.31
Ondo	-7.6077 (0.01)	ln(0)	1,0,6	4653.85
Kebbi	-5.6492 (0.01)	ln(0)	1,0,11	5605.91
Niger	-7.3213(0.01)	ln(0)	1,0,3	6870.56
Yobe	-6.6961 (0.01)	ln(0)	1,0,6	5335.9

With the initial model parameters set, the model for each States time series was estimated within a maximum range between the specified initial for each State (as summarized in Table 8, for which maximum of parameter p,d,q (3,2,*q*) where *q* is based on initial value form denoised data ACF) was used. Forecast of years February 2025 to January 2027 was then carried out using the best performing model. The analysis was done with the aid of the R statistical programming software. The accuracy of these forecast values were analyzed based on low AIC and based on evaluation matrices including minimized values of: BIC, RMSE,MAPE for model selection. Although it was observed in the SARIMAX model that most of the models subjected for selection had similar AIC and BIC, the selection of model was modified to pick model having least BIC, for different groups of models having same AIC. This made the selection of the top 3 best fitted models and finally the selection of the best fitted model achievable. The accuracy of the model was obtained and the best fit ARIMA or SARIMAX model was analyzed. The residual of best models were subjected to testing to determine the statistical properties of the model fitness.

**Table 8:** Analysis of accuracy for best fit ARIMA and SARIMAX model.

State	Model (p,d,q)	AIC	BIC	RMSE	MAPE
Cross Rivers	ARIMA (3,1,3)	4075.86	4109.90	10.74	27.23
	SARIMAX (2,0,2) (0,0,0) 12	4060.43	4094.47	1.99	4.28
Enugu	ARIMA (2,1,4)	3994.30	4027.96	8.81	26.09
	SARIMAX (1,1,4) (0,0,0) 12	4061.8	4095.46	2.25	5.56
Ondo	ARIMA (3,0,5)	4497.25	4545.89	10.03	29.03
	SARIMAX (3,1,6) (0,0,0) 12	4477.69	4531.18	2.47	4.88
Kebbi	ARIMA (2,1,9)	5536.36	5596.47	15.06	17.48
	SARIMAX (3,2,3) (0,0,0) 12	5616.76	5656.83	3.02	4.69
Niger	ARIMA (3,1,3)	6674.35	6710.59	18.40	35.32
	SARIMAX (1,1,0) (0,0,0) 12	8322.54	8338.07	5.79	10.79
Yobe	ARIMA (3,1,5)	5293.39	5336.67	24.45	23.61
	SARIMAX (1,1,2) (0,0,0) 12	5500.87	5524.91	4.99	6.30

**Fig 11:**
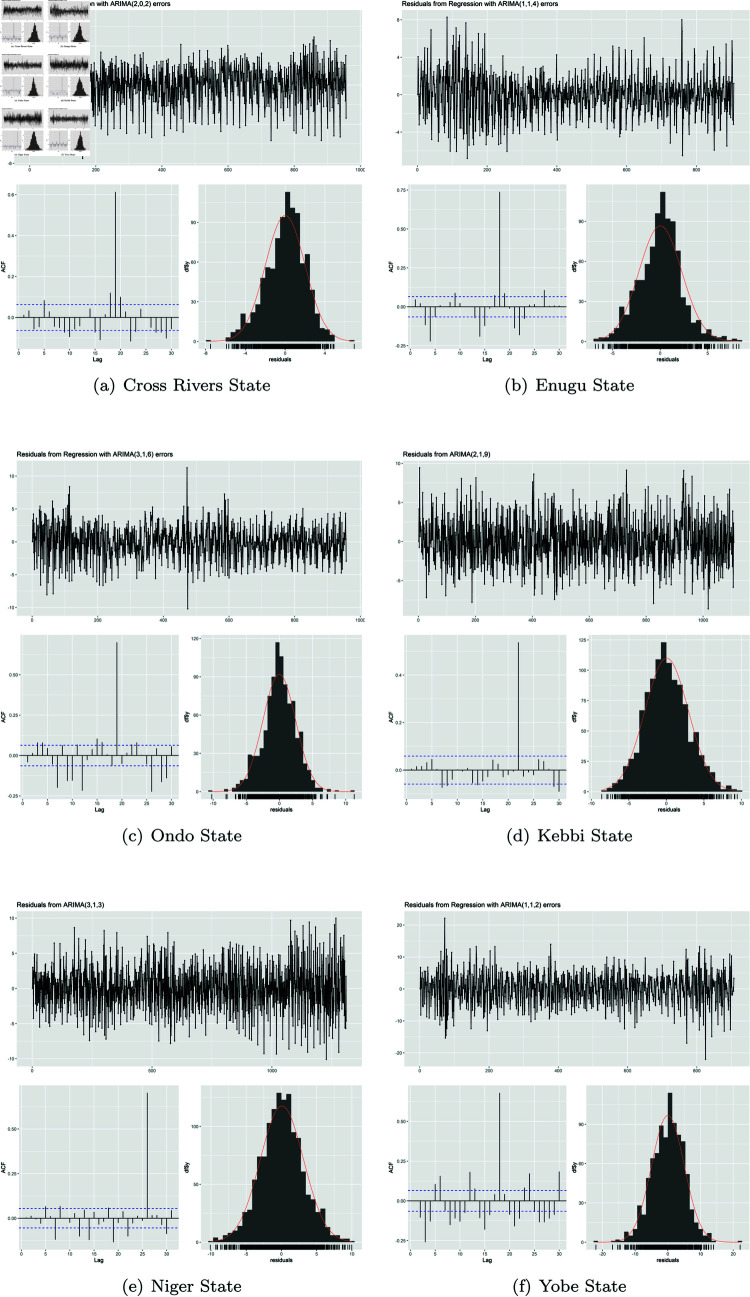
Analysis of residual obtained from best fit ARIMA model.

After carrying out metric evaluation to determine the models’ goodness of fit alongside minimizing complexity of the model, the best model with the lower AIC values in relation to relatively low values of BIC, RMSE and MAPE were chosen. To test the residual of the selected ARIMA model, the study carried out check of the residual (as shown in Fig 11, Table 9) of the selected model for each state. This helped in assessing if the estimated model was able to capture adequate information from the data. This was achieved by testing the residual using the Jarque-Bera test for normality, LM test for ARCH (autoregressive conditional heteroskedasticity) effects, and LB-Q Test for Autocorrelation.

**Table 9:** Result of residual testing of Selected ARIMA/SARIMAX model.

State	JB Test		LM Test (10)		LB-Q (10)	
	jb_stat	p_val	lm_stat	p_val	_stat	p_val
Cross ARIMA (3,1,3)	13.96	9.28e-4	53.16	6.93e-08	15.70	1.08e-1
SARIMAX (2,0,2) (0,0,0) 12	24.18	5.58e-06	35.33	1.09	32.89	2.83
Enugu ARIMA (2,1,4)	31.41	1.50e-07	58.26	7.72e-09	24.87	5.58e-3
SARIMAX (1,1,4) (0,0,0) 12	19.36	6.24e-05	93.24	1.21e-15	73.07	1.12e-11
Ondo ARIMA (3,0,5)	31.75	1.27e-07	73.56	9.05e-12	64.75	4.52e-10
SARIMAX (3,1,6) (0,0,0) 12	22.94	1.04e-05	81.52	2.52e-13	109.16	0
Kebbi ARIMA (2,1,9)	6.14	4.64e-2	17.31	6.77e-2	17.21	6.97e-2
SARIMAX (3,2,3) (0,0,0) 12	0.99	0.60	17.65	6.11e-2	29.47	1.04e-3
Niger ARIMA (3,1,3)	5.28	7.12e-2	83.76	9.15e-14	33.48	2.25e-4
SARIMAX (1,1,0) (0,0,0) 12	2.69	0.26	157.47	1.07e-28	1186.87	0
Yobe ARIMA (3,1,5)	27.92	8.64e-07	47.38	8.01e-07	53.43	6.19e-08
SARIMAX (1,1,2) (0,0,0) 12	21.38	2.26e-05	77.94	1.26e-12	170.89	0

Kebbi and Niger States based on the tests conducted across the respective regions found residuals close to normality (p-value > 0.05), while Cross River, Enugu, Ondo, and Yobe States had the farthest values.

In both ARIMA and SARIMAX, significant ARCH effects (volatility clustering) were observed in Yobe State, reflecting strong fluctuations in MIP incidence. The SARIMAX model for Niger State exhibited notable volatility clustering, suggesting persistent variation in trends over time. Severe heteroskedasticity was identified in Ondo and Enugu States, highlighting considerable variations in MIP incidence, potentially driven by intervention-based fluctuations [80]. Cross River State displayed moderate signs of volatility clustering, with its SARIMAX model showing substantial deviations in residual behavior. Kebbi State, on the other hand, demonstrated the most stable trend, with the SARIMAX model exhibiting residuals closest to normality, indicating minimal volatility clustering. Overall, the results confirm that Northern states (especially Yobe and Niger) show strong volatility, reinforcing the patterns observed in malaria prevalence studies, while Southern states display intervention-driven fluctuations with Cross River showing moderate variability.

Furthermore, all regional residuals exhibited strong autocorrelation, persisting even after multiple model adjustments aimed at improving fit [26]. In Niger State, autocorrelation in the SARIMAX model was significantly greater than in ARIMA, highlighting persistent dependencies in malaria incidence trends. Kebbi State’s SARIMAX residuals, while displaying structured patterns, suggested potential underlying malaria transmission dynamics that remain unexplained [24].

Although SARIMAX results indicated no clear seasonal component, however similar to ARIMA, comparisons between the two models reveal variations in statistical properties across regions, influenced by differing exogenous factors [42]. While no strong seasonality was detected, fluctuations in malaria incidence and residual variability may be driven by external influences such as climate conditions, healthcare accessibility, intervention effectiveness, or migration patterns [82]. It is observed that Kebbi and Niger states exhibited the most stable residuals, suggesting minimal seasonal effects, whereas Yobe, Enugu, and Ondo demonstrated significant heteroskedasticity, potentially due to intervention-based or environmental fluctuations.

Unresolved autocorrelation indicates that malaria treatments could be better timed to align with recurring transmission cycles [83]. In line with the research objective of understanding the variability of MIP across different Nigerian states, the comparative findings from time series analysis highlight important regional differences in malaria incidence, helping to inform more effective malaria control strategies targeted at pregnant women.

All SARIMAX models offered deeper insight into MIP transmission cycles and incidence trends across regions, although for Kebbi State, the simpler ARIMA model provided a better fit. This implied that: (i) in Cross River State, seasonality may not be a dominant factor, but other exogenous variables (such as environmental or intervention effects) are needed to improve model optimization; (ii) malaria cases fluctuate due to external factors in Enugu; therefore, it is important to incorporate weather trends, intervention coverage, and socioeconomic factors when building epidemiological models; (iii) Ondo State indicates MIP incidence was no stable; thus models would work best if meaningful exogenous factors, such as seasonal interventions are included; (iv) Niger State exhibited strong seasonality or intervention-driven fluctuations; therefore, climatic factors should be accounted for; and (v) MIP cases followed strong cycles in Yobe State, confirming the presence of consistent transmission cycles that influence malaria trends in the region.

**Fig 12:**
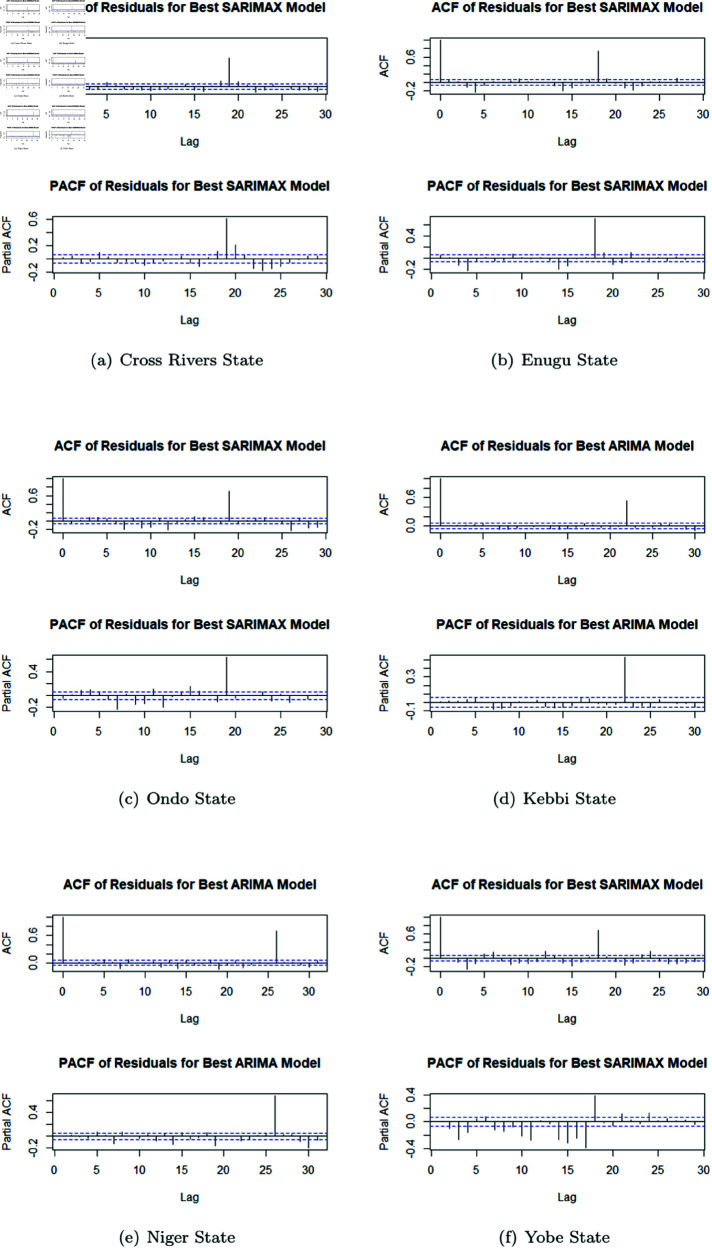
ACF and PACF of Residual obtained from best fit ARIMA model.

From the plotted ACF (as shown in Fig 12, it could be concluded that the residuals’ ACF plots show good fitness of models. The study thus proceeded with the forecast. It should be mentioned that a comparison of Niger State SARIMAX forecast(2900 cases) and its ARIMA forecast (210 cases). This demonstrates that the SARIMAX model predictions experience a significant increase in cases in Niger which could be as a result of intervention decay effects, potentially leading to an overestimation of the malaria burden by 2027. Also, sensitivity to exogenous variables may be exaggerating the prediction, as demonstrated by comparative study with ARIMA, which calls for careful interpretation of long-term forecasts. Additional intervention modeling improvements, including modifying decay parameters could increase prediction stability and guarantee realistic scenario planning. Also, prediction stability and accuracy may be increased by optimizing exogenous inputs or investigating alternative modeling techniques, such as hybrid epidemiological models that integrate climate and intervention dynamics. These are areas we intend to apply in further research. Thus using SARIMAX model for all other States and ARIMA model for Kebbi and Niger State was adopted. This approach ensures the best statistical fit and predictive accuracy based on findings.

**Fig 13:**
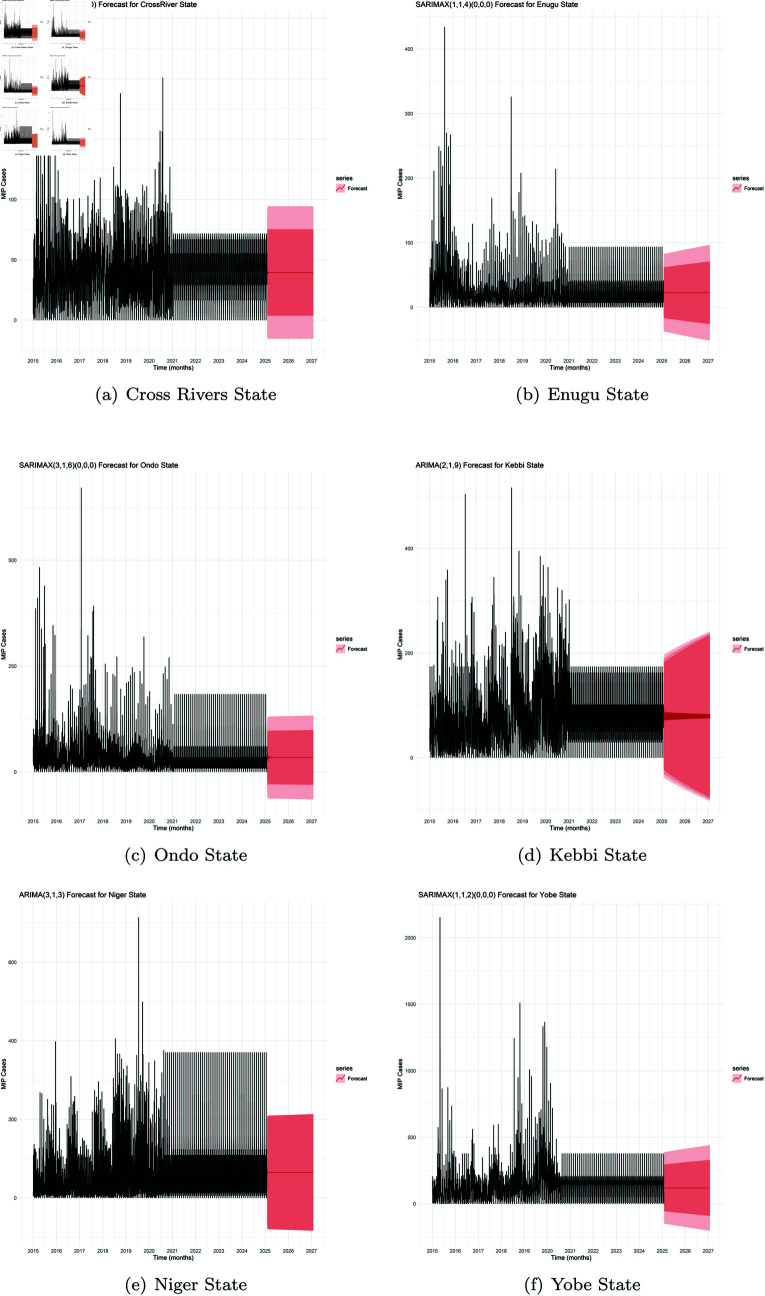
ARIMA model and forecast values in the 95% confidence interval.

The forecast results (as shown in Fig 13) represented each regions’ expected number of malaria cases for future periods until January 2027, based on historical data.

It was observed in these regions, that the actual number of cases estimated within a 95% confidence interval could reach possible cases by the end of 2027, as high as: 90 in South-South (Cross Rivers State), 95 in the South-East (Enugu), 125 in South-West (Ondo State), 240 in North-West (Kebbi State), 210 in North Central (Niger State) and about 400 in North-East (Yobe State), respectively.

These forecast results are extrapolated and should be interpreted with caution due to lack of original post-2020 inputs for MIP cases. Based on the facts available, to reduce malaria cases and achieve elimination in the various regions, there is need to double efforts in the control interventions deployed while targeting the seasonal peak periods of MIP cases peculiar for each region. More attention should also be given to prioritizing the North-East and North-West so as to reduce the population of pregnant women being prone to malaria in the nation.

### Implication for malaria control efforts in Nigeria

Based on the above results, these findings reveal significant regional variability in malaria in pregnancy (MIP) incidence, emphasizing the need for targeted interventions across Nigeria’s six geopolitical zones. South-South (Cross River State) peak incidence in 2017 and 2019 suggests potential gaps in malaria prevention strategies, such as intermittent preventive treatment in pregnancy (IPTp) coverage and insecticide-treated net distribution. While malaria control efforts were ongoing, climatic conditions, river flooding, and low accessibility to antenatal care services may have contributed to increased MIP cases. Moderate residual autocorrelation suggests some underlying patterns that remain unresolved in transmission dynamics, reinforcing the need for better maternal health programs and expanded malaria surveillance to mitigate future spikes.

South-East (Enugu State) with the lower troughs recorded in 2017, malaria incidence among pregnant women appears more stable compared to other regions. This stability may stem from better urban healthcare access, higher IPTp coverage, and lower transmission intensity. However, significant heteroskedasticity observed in residuals indicates persistent volatility in malaria trends, pointing to the need for socioeconomic interventions and improved record-keeping at local health centers to enhance malaria burden assessments.

South-West (Ondo State) strong autocorrelation and ARCH effects observed in residuals suggest periodic outbreaks and intervention-driven fluctuations, highlighting that seasonal interventions directly influence transmission cycles. The presence of short-term cycles confirms the instability of MIP incidence, requiring enhanced monitoring of intervention effectiveness, IPTp adherence, and health facility utilization to minimize malaria variability.

North-West (Kebbi State) malaria transmission displays long-term coherence trends, reinforcing endemicity and seasonality-driven outbreaks. ARIMA performed better than SARIMAX, but persistent autocorrelation suggests some unresolved malaria transmission dynamics. Improved intervention timing and adherence to IPTp uptake among pregnant women should be prioritized, alongside expanded community-based malaria monitoring to support early detection of seasonal surges.

North-Central (Niger State) exhibits strong ARCH effects, indicating hidden transmission patterns possibly linked to seasonality or intervention coverage gaps. While MIP incidence appears stable, short-term fluctuations suggest the need for climate-sensitive forecasting models to adapt malaria response efforts based on rainfall trends, temperature variations, and vector density cycles. Projected peaks in MIP cases by 2027, the data highlight an urgent need for aggressive malaria control strategies

North-East (Yobe State) with projected peaks in MIP cases by 2027, also highlight an urgent need for aggressive malaria control strategies. Strong cyclic trends and autocorrelation indicate malaria seasonality as a primary transmission driver, suggesting interventions need to align with peak cycles rather than rely on static treatment schedules. Expanded healthcare access, climate adaptation measures, and localized malaria surveillance will be crucial in reducing MIP burden.

The findings emphasize the necessity of region-specific interventions, particularly in high-burden states.

South-South and South-East require healthcare accessibility improvements, with Cross River exhibiting moderate residual patterns and Enugu showing strong volatility in malaria incidence.

North-Central and North-West need better integration of seasonality-based forecasting tools, particularly in Niger, where strong ARCH effects were detected, and Kebbi, which requires improved intervention timing despite ARIMA’s better fit.

North-East demands urgent malaria control measures, reinforcing the need for strengthened antenatal malaria monitoring and targeted intervention deployment.

### Study limitations

This study provides valuable insights into malaria in pregnancy (MIP) incidence trends across Nigeria’s six geopolitical zones. By applying wavelet coherence and time series modelling, regional variations were identified, and future transmission patterns were forecasted. There are several limitations that must be acknowledged to ensure proper interpretation and application of these findings. Data collection biases in health facility-based reporting may have underestimated true malaria incidence, as cases occurring after the COVID-19 pandemic (post-2021 data) were not captured. Also, variations in data quality across regions and disparities in record-keeping infrastructure may have influenced accuracy and model reliability.

The use of ARIMA and SARIMAX models assumed stationarity, yet malaria transmission cycles may be influenced by nonlinear climatic trends, potentially affecting model precision. Future research we intend to embark hybrid epidemiological models incorporating machine learning techniques to improve prediction accuracy and adaptation to dynamic transmission factors. This will help us achieve increased prediction stability and accuracy were hybrid epidemiological models that integrate climate and intervention dynamics will be analysed. These are areas we intend to apply in further research. Wavelet coherence analysis successfully identified short- and long-term malaria transmission periodicities, but it did not point out causal mechanisms, indicating the need for further epidemiological studies to establish direct links between interventions, environmental factors, and transmission peaks.

Potential overfitting in regional models was observed, particularly through ARCH effects and autocorrelation persistence, suggesting that unaccounted exogenous factors such as IPTp intervention coverage, climatic influences, or socioeconomic disparities may be driving MIP transmission trends. These aspects should be investigated in future studies to refine forecasting models and intervention strategies.

Despite these limitations, this study provides a comprehensive analysis of MIP transmission trends, offering evidence-based recommendations for malaria intervention strategies tailored to regional transmission patterns.

## Conclusion

Malaria in pregnancy (MIP) remains a significant public health concern in Nigeria, contributing to maternal morbidity, adverse birth outcomes, and increased neonatal mortality. The high rate of malaria-related deaths in pregnant women poses an urgent challenge, as maternal fatalities and stillbirths represent irrecoverable losses to families and communities.

This study successfully addressed the question of long-term trends in MIP incidence over the past decade (Jan 2015—Jan 2025). Findings revealed substantial variability in malaria incidence across regions, such as the South-South (Cross River State) reaching its highest peak in MIP cases in 2017, while the South-East (Enugu) experienced its lower trough in the same year. The highest observed peaks in South-South, South-East, and South-West (6.5 change in malaria incidence) were lower than the lowest recorded troughs in all the Northern regions. This reinforces the need for cost-effective, region-specific malaria interventions, considering the distinct transmission dynamics and environmental influences in each geopolitical zone.

The second research question was addressed by analyzing MIP case trends compared to general malaria case (MC) trends across different regions. Findings revealed strong cross-correlations over an extended period at high frequency cycles, with North-West (Kebbi) exhibiting the strongest correlation, followed by North-Central (Niger), North-East (Yobe), and South-West (Ondo). In contrast, South-South (Cross River) and South-East (Enugu) showed weaker but consistent correlation patches at similar frequencies. The peak months recorded for MIP cases recorded were: South-South (3rd week June & beginning of September), South-East (1st week July), South-West (2nd weeks in July and August), North-West (1st week October), North Central (Mid August) and North-East (4th weeks in October), respectively. The seasonal persistence of MIP cases and their strong correlation with general malaria transmission trends highlights malaria’s endemicity in Nigeria. Also, while the MIP population is proportionally smaller compared to the general population, the increase in maternal mortality due to malaria has far-reaching effects on public health and neonatal survival, demanding strategic interventions specific to pregnant women. Notably, peak MIP cases often precede peak general malaria incidence, emphasizing the unique vulnerability of pregnant women and the need for timely intervention strategies tailored to their specific risk factors.

Forecasting results confirmed significant heterogeneity in MIP trends, with each state requiring a customized SARIMAX model approach. Persistent autocorrelation in multiple models suggests that seasonality and external environmental factors continue to influence malaria transmission cycles. In particular, Cross River State exhibited strong ARCH effects, reinforcing the role of seasonal fluctuations in malaria burden. Future projections indicate potential peaks in MIP cases by 2027 in the Northern regions emphasizing the urgent need to prioritize interventions in these high-burden areas.

Pre-analysis of data obtained from NMDR has provided valuable insights into how MIP cases compare with general MC cases over time (2015–2024), identifying seasonal variations and potential future trends. Findings clearly indicate strong regional variability in malaria transmission patterns, emphasizing that each geopolitical zone experiences unique transmission dynamics. Given Nigeria’s endemic malaria status,improved monitoring and coordinated supervision of MIP data collection and reporting are crucial for accurate modelling and prediction analysis to support effective intervention strategies.

The Nigerian government has implemented multiple malaria control strategies, but eliminating malaria will require more targeted, region-specific approaches that address the unique variability observed in MIP cases. This study confirms that malaria incidence differs significantly by region, with Northern states consistently recording higher cases. To achieve the Nigeria Malaria Strategic Plan (NMSP) 2021–2025 goal of reducing malaria prevalence to below 10%, informed regional strategies must be prioritized. Applying tailored intervention approaches for MIP control will enhance decision-making for intervention deployment, improve planning efforts, and optimize malaria burden reduction nationwide.

This study faced data limitations, particularly missing MIP records post-2021, which may affect forecast reliability. Also while ARIMA and SARIMAX models assumed stationarity, unaccounted nonlinear climatic trends and external intervention factors may have influenced malaria transmission dynamics. Also, persistent autocorrelation and ARCH effects indicate unaccounted exogenous factors—such as IPTp coverage, climate variability, and socioeconomic disparities—that may be driving MIP transmission trends. Also the sensitivity of SARIMAX model to exogenous factors among other limitations earlier mentioned, highlight the need for deeper epidemiological and machine learning studies to achieve better prediction results. This is an area of further study to better prediction stability and accuracy to optimize exogenous inputs when integrating climate and intervention dynamics.

It is recommended that to improved malaria control, strengthening of malaria record-keeping in primary health facilities, ensuring consistent data reporting and quarterly monitoring is crucial. Improved IPTp coverage and targeted interventions and emphasis for ANC compliance, especially in high-risk regions like in Northern regions should be campaigned, given that MIP trends suggest early warning signs of malaria surges. Enhanced malaria surveillance systems, incorporating real-time data tracking to optimize intervention timing and effectiveness are also needed. Prioritizing seasonal intervention efforts is a must, to align malaria control programs with peak transmission months in each region. Integrating forecast-driven malaria control measures to significantly reduce MIP burden and help Nigeria achieve its National Malaria Elimination Programme (NMEP) objectives.
